# Emergence of robust growth laws from optimal regulation of ribosome synthesis

**DOI:** 10.15252/msb.20145379

**Published:** 2014-08-22

**Authors:** Matthew Scott, Stefan Klumpp, Eduard M Mateescu, Terence Hwa

**Affiliations:** 1 Department of Applied Mathematics, University of WaterlooWaterloo, ON, Canada; 2 Max Planck Institute of Colloids and InterfacesPotsdam, Germany; 3 Department of Physics and Center for Theoretical Biological Physics, University of CaliforniaSan Diego, La Jolla, CA, USA; 4 Institute for Theoretical Studies, ETH ZurichZurich, Switzerland

**Keywords:** growth control, metabolic control, phenomenological model, resource allocation, synthetic biology

## Abstract

Bacteria must constantly adapt their growth to changes in nutrient availability; yet despite large-scale changes in protein expression associated with sensing, adaptation, and processing different environmental nutrients, simple growth laws connect the ribosome abundance and the growth rate. Here, we investigate the origin of these growth laws by analyzing the features of ribosomal regulation that coordinate proteome-wide expression changes with cell growth in a variety of nutrient conditions in the model organism *Escherichia coli*. We identify supply-driven feedforward activation of ribosomal protein synthesis as the key regulatory motif maximizing amino acid flux, and autonomously guiding a cell to achieve optimal growth in different environments. The growth laws emerge naturally from the robust regulatory strategy underlying growth rate control, irrespective of the details of the molecular implementation. The study highlights the interplay between phenomenological modeling and molecular mechanisms in uncovering fundamental operating constraints, with implications for endogenous and synthetic design of microorganisms.

## Introduction

Cell growth requires protein synthesis, carried out by ribosomes which polymerize amino acids into polypeptide chains. The efficient conversion of environmental nutrients into amino acids and incorporation of amino acids into stable protein mass is of central importance to enteric bacteria such as *Escherichia coli* which live in rapidly varying environments. Many of the biochemical details of bacterial metabolism and protein synthesis have been elucidated over the past 50 years (White *et al*, 2011), and it is clear that at the molecular level, synthesis, degradation and regulation are implemented via complex interconnected networks, governed by kinetics that depend nonlinearly on reactant concentrations (Karr *et al*, 2012). Nevertheless, at the physiological level, simple empirical relations appear; these are known as “growth laws” (Scott & Hwa, 2011).

**Figure 1:**
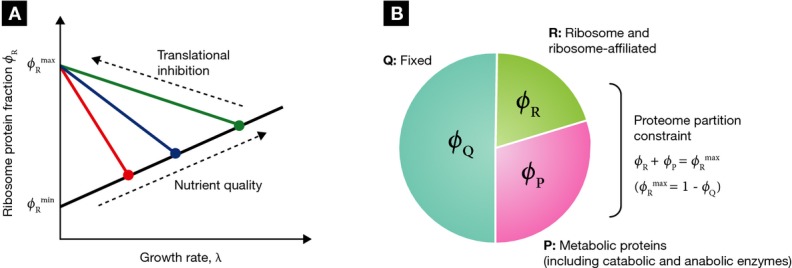
Linear growth relations and minimal partitioning of the proteome (A) Empirical relations between the ribosomal protein fraction and growth rate in exponentially growing *Escherichia coli*. Under changes in nutrient quality (filled symbols) or translational capacity (colored lines), the ribosomal protein fraction ϕ_R_ is a linear function of the growth rate λ. (B) The growth relations in (A), along with data on metabolic proteins responsible for coordinating carbon and nitrogen assimilation (You *et al*, 2013), suggest that a minimum partitioning of the proteome consists of three protein fractions (Scott *et al*, 2010): a growth rate-independent fraction ϕ_Q_, a fraction including ribosome-affiliated proteins ϕ_R_, and a metabolic fraction ϕ_P_ containing the remainder, including catabolic and anabolic enzymes. The growth rate dependence of the ribosome and metabolic proteins are constrained by the partitioning so that 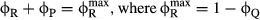.

For example, under balanced exponential growth, the macromolecular composition of *Escherichia coli* is correlated simply with the growth rate of the culture, largely independent of the specific nutrients in the growth medium (Schaechter *et al*, 1958; Cooper, 1993; Bremer & Dennis, 1996; Scott *et al*, 2010). In batch culture, bacterial growth rate can be modulated through the composition of the growth medium. By varying the quality of the supplied nutrients, (for example, by changing the carbon source or adding a variety of amino acids, nucleosides and vitamin supplements), the doubling time can be easily varied from 20 min up to several hours. Under these conditions, with growth rate modulated by nutrient quality, the ribosomal protein fraction increases linearly with the growth rate (black line, Fig 1A). Conversely, when the medium composition is fixed and protein translation is impaired through antibiotic treatment, the reduction in growth rate is accompanied with a linear increase in ribosomal protein fraction (colored lines, Fig 1A).

These two empirical observations relating growth rate and ribosomal content can be combined with a coarse-grained partitioning of the proteome to provide a predictive model for the response of the bacterium to physiological perturbations (Scott & Hwa, 2011; Klumpp & Hwa, 2014). In the simplest case, the total proteome is partitioned into a growth rate-independent fraction that may include negatively autoregulated housekeeping genes (Klumpp *et al*, 2009), and growth rate-dependent fractions, one for ribosomal and other translational proteins, and one for metabolic proteins, including transporters and catabolic and anabolic enzymes (Scott *et al*, 2010; Scott & Hwa, 2011). This partitioning results in a constraint on the growth-dependent allocation of these fractions; if the ribosomal protein fraction is increased, it must do so at the expense of reducing the metabolic protein fraction (Fig 1B). The resulting phenomenological framework that comes from combining the empirical growth laws with a coarse proteome partitioning has been used to predict successfully the burden of heterologous protein expression (Scott *et al*, 2010), to elucidate key molecular interactions underlying carbon catabolite repression (You *et al*, 2013), to reveal intrinsic feedback effects governing drug/drug resistance interaction (Deris *et al*, 2013), and to predict how biosynthetic pathways balance enzyme cost with product demand (Li *et al*, 2014). Yet critical features of the underlying regulation that give rise to the growth laws remain unclear. Specifically, what molecular mechanisms are responsible for the allocation of cellular resources that guarantee optimal growth irrespective of the nutrient environment and how do simple empirical relationships emerge from complex metabolic networks?

Here, we show that the growth laws originate from constraints on the supply flux of amino acids and their consumption through protein synthesis. Subject to these flux constraints, we demonstrate that there is an optimal partitioning of cellular resources for a given growth environment that maximizes these fluxes at steady state and hence maximizes growth rate.

Our analysis reveals the central role played by a pair of interlocked regulatory loops. The first one is a feedback loop on amino acid supply by end-product inhibition that ensures the stability of the steady state (Savageau, 1977) and effectively isolates sensing, adaptation and processing of amino acid supply from the protein synthesis machinery. The second loop is a “supply-driven activation” feedforward loop, which controls amino acid flux and consequently the rate of protein synthesis, by responding to any mismatch between amino acid supply and consumption. Supply-driven activation is a simple mechanism to balance amino acid flux through protein synthesis and central metabolism and may be a preferred method of maintaining flux balance in exponentially growing organisms. In a wider context, our analysis provides an example of empirical laws in biology being used to infer underlying robust regulation. This general approach of using phenomenology to constrain mechanism should be broadly applicable to reveal proteome-wide regulatory strategies in other exponentially growing organisms, including eukaryotic microbes and tumor cells.

## Fundamental constraints on amino acid flux

The two empirical growth laws described above can be expressed with the following relations. First, when growth rate is changed by modifying the nutrient composition of the medium, the mass fraction of ribosomal proteins ϕ_R_ varies linearly with the growth rate λ and has a positive slope (1/γ),


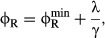
(1)

(black line, Fig 1A). Second, when growth rate is changed by inhibiting protein synthesis (for example, by antibiotics), the mass fraction of ribosomal proteins ϕ_R_ remains linearly dependent on the growth rate λ, but now with a negative slope (−1/ν),


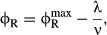
(2)

(colored lines, Fig 1B). The empirical parameters 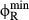 and 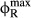 are approximately growth medium independent and set the limits on the ribosomal protein fraction during exponential growth. The empirical parameter γ is proportional to the *in vitro* protein translation rate (Scott *et al*, 2010; Klumpp *et al*, 2013), and ν correlates with the nominal growth rate of the strain in a given medium in the absence of antibiotics (Scott *et al*, 2010). The parameters γ and ν are therefore referred to as the translational and nutritional efficiency, respectively.

In what follows, our objective is to determine the regulatory mechanisms that give rise to the empirical growth laws (Fig 1A). To connect the phenomenological relations with underlying regulation, we first provide a review of molecular interpretations of the parameters γ and ν appearing in the two empirical laws expressed in equations (1 and 2), as well as the constraints linking protein synthesis, metabolism, and growth.

## Protein synthesis

The processes involved in cellular adaptation and growth are complex; to simplify the system as much as possible, we will consider only exponential growth. In this balanced state of growth, every constituent of the cell doubles at the same rate. For cells doubling once-per-hour, for example, the total DNA content of the cell must double every hour, but so, too, must the total RNA content, the total protein content, and so on for all molecular species in the cell. There is no net accumulation of any one constituent, and daughter cells are indistinguishable from their mothers.

Growth at constant exponential rate imposes strong constraints on how the cell allocates its internal resources, particularly the protein synthesis machinery. In fact, the first empirical growth law that ribosomal protein fraction is an increasing linear function of growth rate when growth rate is modulated by nutrient quality [as expressed symbolically by equation (1)] follows simply from constraints imposed by exponential growth (Maaløe, 1979).

In exponential growth, the entire cellular content increases at the same rate, including the total protein mass *M*. Neglecting protein turnover, exponential protein mass accumulation is written as,


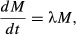
(3)

where λ is the exponential growth rate. But protein mass accumulation is maintained by a number of ribosomes 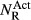 actively involved in protein biosynthesis, all translating at an averaged rate *k* per ribosome,


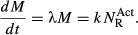
(4)

Not all ribosomes are active; there will be a number of ribosomes 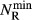 not participating in protein synthesis. Contributions to this inactive subpopulation include ribosomes in search of mRNA ribosome binding sites (Scott *et al*, 2010), ribosome recycling (Pavlov *et al*, 1997), and ribosomes paused awaiting charged tRNA (Klumpp *et al*, 2013). Writing the rate of protein mass accumulation in terms of the total number of ribosomes *N*_R_, from equation (4),


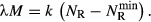
(5)

The total mass of ribosomal proteins is denoted by 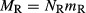, where 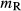 is the mass per ribosome along with its cohort, that is, all proteins co-regulated with ribosomal proteins such as initiation factors, elongation factors, etc. (Howe & Hershey, 1983; Bremer & Dennis, 1996). The mass fraction of ribosomal proteins is denoted 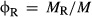, so that dividing equation (5) by total protein mass *M* yields,


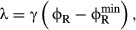
(6)

with the elongation rate now expressed as a translational efficiency in units of 1/time, 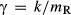. Equation (6) results in the empirical linear relation equation (1), as long as γ and 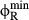 remain constant as the growth rate is varied. This appears to be the case when growth rate is modulated by changes in the nutrient composition of the growth medium.

## Amino acid flux

To maintain the protein biosynthesis required for growth, a steady influx of amino acids must be supplied to the ribosome to feed the elongating peptide chains. As above, exponential growth imposes strong constraints on amino acid flux. The dynamics of the free amino acid pool within the cell is determined by the amino acid influx rate on one hand and by their incorporation into proteins on the other hand. In media with amino acids supplied, influx is limited by the efficiency and the relative abundance of proteins involved in amino acid supply such as transport proteins. These transport proteins are part of the fraction of the proteome that is involved in metabolism and nutrient assimilation. Consequently, using the constraint that the sum of the mass fraction of ribosomal proteins and metabolic proteins remains constant, any increase in metabolic protein fraction to increase amino acid supply must necessarily decrease ribosomal protein fraction, and thereby decrease amino acid consumption through protein synthesis. As we derive below, this balance of amino acid flux subject to the proteome partitioning constraint results in the second empirical growth law, equation (2).

In a given growth environment, we assume that protein synthesis is limited by the supply flux of one of the amino acids (or a small group of amino acids), and denote that growth-limiting amino acid pool by a single coarse-grained entity of total mass *M*_*a*_. Under the assumption that protein turnover is negligible, the dynamics is governed by


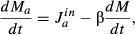
(7)

where 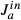 is the amino acid influx rate and β is the fraction of translation events consuming the growth-limiting amino acid, given by the frequency of the growth-limiting amino acid used in proteins (e.g. 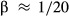 if all amino acids are present in equal frequencies). It is convenient to normalize equation (7) by the total protein mass *M* in order to connect with the protein mass accumulation equation (6),


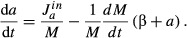
(8)

We will refer to *a* = *M*_*a*_/*M* below as the (free) “amino acid” level. It is the mass fraction of the collective growth-limiting amino acid variable and is proportional to the intracellular concentration (Box 1)—using an average molecular weight of 110 Daltons per amino acid, a concentration of 1 mM corresponds to a mass fraction of about 3.8 × 10^−4^. From Supplementary Table S1, typical amino acid concentrations are in the 1–10 mM range, with corresponding mass fraction *a* < 5 × 10^−3^, so that 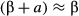. In steady state, there is no net change in the amino acid pool, 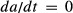, and the amino acid dynamics simplify to the algebraic constraint,


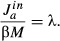
(9)

**Box 1 – From mass fraction to concentration**

Throughout, our focus will be on the protein fraction devoted to ribosomal and metabolic proteins, and how the total proteome is partitioned between these two classes to maximize the rate of protein synthesis and cell growth. In terms of the proteome fraction, it is straightforward to invoke constraints linking these two protein classes (Fig 1B). Nevertheless, in large-scale metabolic models, it is more typical to use units of concentration in place of mass fraction. From the proportionality between the total protein mass and the cell's dry mass (Bremer & Dennis, 1996), and the constancy in the cell density across nutrient conditions (Kubitschek *et al*, 1984), a quantity normalized by the total protein mass is a proxy for the intracellular concentration, for example, ϕ_R_ is proportional to the ribosome concentration. It has been previously estimated that the conversion factor from concentration *c*_*i*_ to mass fraction, ϕ_*i*_ = *σc*_*i*_, is approximately *σ* = 3.8 × 10^−7^ *μ*M × *N*_*aa*_ where *N*_*aa*_ is the number of amino acids in the protein of interest (Klumpp *et al*, 2013). For a typical protein of 330 amino acid residues, a mass fraction of 0.1% corresponds to about 8 μM [see also Milo (2013)].

In media with amino acids supplied, influx is limited by transport, and transport proteins share the same growth rate dependence as other metabolic proteins; we denote by *η*_*a*_ the fraction of metabolic proteins that are used to transport the amino acid. For a total metabolic protein mass *M*_P_, the flux can be written as,


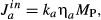
(10)

where *k*_*a*_ is a proportionality constant that characterizes the efficiency of the transporters. Dividing through by total protein mass,


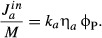
(11)

Thus, in our model, it is the rate of amino acid influx that is proportional to the mass fraction of metabolic protein ϕ_P_, and not the amino acid level itself that is proportional to ϕ_P_ as has been assumed in other models of optimal proteome allocation (Zaslaver *et al*, 2009). Substituting equation (11) into equation (9), the amino acid flux equation becomes,


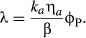
(12)

The advantage of expressing protein abundance in terms of mass fraction is that we can invoke a simple proteome partitioning constraint, 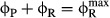 (Fig 1B), and re-write the amino acid flux in terms of ribosomal protein fraction alone,


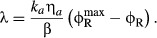
(13)

Equation (13) gives rise to the observed negative linear relation equation (2) with the empirical nutritional efficiency *ν* identified as


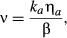
(14)

whenever changes in the growth conditions are such that this nutritional efficiency is left unchanged. Experimentally, this was done in (Scott *et al*, 2010) by adding sub-lethal concentrations of translation-inhibiting antibiotics to the growth medium for a fixed nutrient composition, which has the primary effect of reducing the translational efficiency γ without significantly affecting *ν*. The interpretation provided by equation (13) is that nutritional efficiency *ν* is a growth medium-dependent phenomenological parameter that includes the relative expression level *η*_*a*_ and the efficiency *k*_*a*_ of amino acid uptake. Regulation of the nutritional efficiency can be implemented through changes in efficacy *k*_*a*_ (e.g. allosteric inhibition) or protein expression level *η*_*a*_ (e.g. transcriptional repressors, attenuation, etc). Large nutritional efficiency *ν* corresponds to a nutrient environment for which the organism can sustain a high amino acid supply flux while keeping the mass fraction of supply proteins ϕ_P_ low. In minimal media without amino acids in the environment, the amino acid supply flux is given by the rate of amino acid biosynthesis and a relation similar to equation (13) is obtained in that case as well (see Supplementary Fig S1). In the case where the quantity of the nutrient is limited, Monod kinetics emerge naturally from this formulation (Box 2 and Supplementary Fig S1).

**Box 2 – Monod kinetics**

For transport-limited supply of the growth-limiting amino acid a, the efficacy *k*_*a*_ can be written in a Michaelis form,

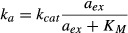
(24)

where *a*_*ex*_ is the external concentration, and *k*_*cat*_ and *K*_*M*_ characterize the speed and affinity of the transporter. With substitution into equation (13) (with 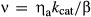), and using equation (6) to eliminate ϕ_R_, a Monod relation for the growth rate λ is obtained (Monod, 1949),

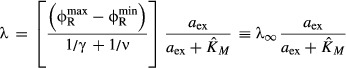
(25)

where *λ*_∞_ is the growth rate in limit *a*_*ex*_ → ∞. The apparent Michaelis constant, 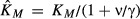 carries an explicit growth medium dependence through the translation efficiency γ and the nutrient efficiency *ν*. A similar expression emerges from growth limited by the transport of simple sugars (the case originally studied by Monod); see Supplementary Fig S1.

## Growth rate maximization

**Figure 2:**
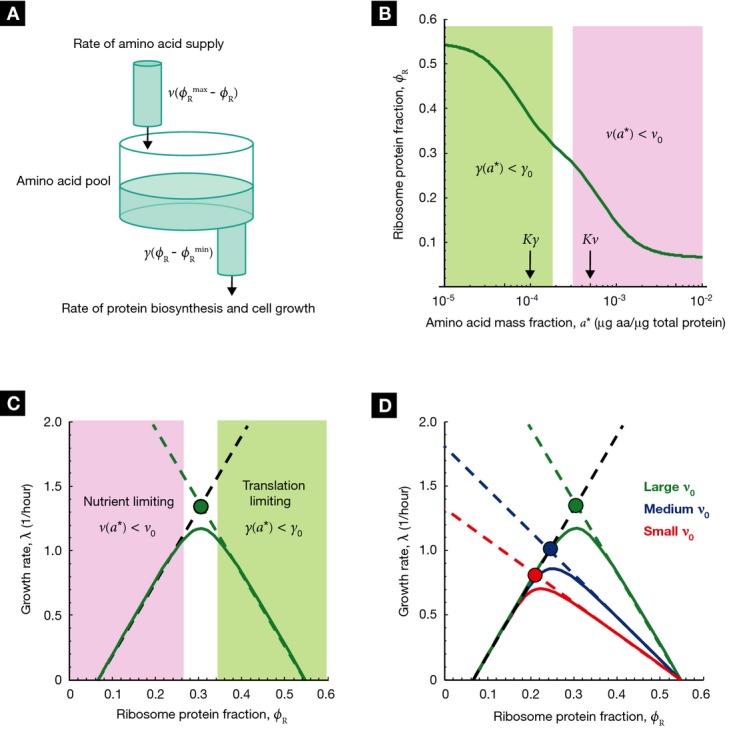
Amino acid flux balance and growth rate maximization (A) In exponential growth, the amino acid consumption rate via protein synthesis, 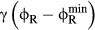, must balance the supply rate via transport and biosynthesis, *ν*ϕ_P_ [equation (12)], to maintain a constant amino acid pool size. Using the proteome partitioning constraint that ribosomal protein fraction ϕ_R_ and metabolic protein fraction ϕ_P_ sum to a constant, 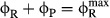 (Fig 1B), the supply rate can be written as 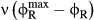. The cell then must regulate the ribosomal protein fraction ϕ_R_ to both balance and maximize the flux through the system. (B) The ribosomal protein fraction ϕ_R_ determines the steady-state amino acid level a* (green solid line) and consequently the growth rate λ [equation (17)], when the amino acid flux is balanced. (C) The growth rate λ (green solid line) exhibits a unique maximum corresponding to an optimal size of the ribosomal protein fraction ϕ_R_. The upper bound on the growth rate maximum occurs when the translational efficiency 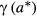 and nutritional efficiency 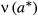 are both maximal for a given nutrient environment, 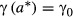 and 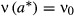 (filled circle). (D) The optimal size of the ribosomal protein fraction ϕ_R_ depends upon the growth environment (filled circles), illustrated here by a change in the nutrient quality of the medium: poor nutrient *ν*_0_ = 2.5/h (red solid line), good nutrient *ν*_0_ = 3.3/h (blue solid line), and rich nutrient *ν*_0_ = 5.8/h (green solid line). Dashed lines correspond to the empirical relations shown in Fig 1A, 
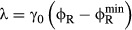
 (black dashed line) and 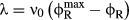 (colored dashed lines). The amino acid level for efficient peptide elongation *K*_*γ*_ = 10^−4^, and the level to trigger negative feedback inhibition of amino acid supply *K*_*ν*_ = 5*K*_γ_ = 5 × 10^−4^. The remaining parameters are *γ*_0_ = 5.9/h, 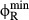 = 0.07 and 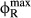 = 0.55 (Scott *et al*, 2010).

The constraints on amino acid flux, and its relation to growth, are depicted schematically in Fig 2A. In steady-state exponential growth, the rate of amino acid supply must be balanced by the rate of amino acid consumption through protein synthesis to ensure that there is no net change in the amino acid pool [equation (12)]. Furthermore, in exponential growth the rate of protein synthesis is synonymous with the rate of bacterial growth [equation (6)], so the cell is faced with the twin objectives of balancing and maximizing the amino acid flux in order to maximize growth rate.

For a given translational efficiency γ and the nutritional efficiency *ν* (as determined by the growth medium), the organism must choose the ribosomal protein fraction ϕ_R_ that balances the amino acid flux. Mechanistically, the way that this is done is to use the amino acid pool size as a sensor for flux mismatch: if the amino acid pool size increases, that is indicative of supply exceeding demand, so regulation increases the ribosomal protein fraction ϕ_R_ to increase demand via protein biosynthesis and simultaneously decrease supply via the proteome constraint 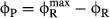 (Fig 1B). We call this control strategy “supply-driven activation” of ribosomal protein synthesis. As we will show below, this strategy together with a number of auxiliary conditions is sufficient for the cell to achieve flux balance and maximal growth rate over a range of growth conditions. But we will first discuss growth limitations that can in principle arise when amino acid pool size is very large or very small and show how these inefficient limiting cases are avoided by the regulatory mechanisms of the cell. We will then describe the molecular implementation of supply-driven activation in the regulation of ribosomal protein synthesis and show how it achieves growth rate maximization.

### Stabilizing amino acid flux

In the limit where amino acid pools drop low enough that tRNA charging becomes limiting, the protein translation rate will decrease (Elf & Ehrenberg, 2005). Although the cell has evolved strategies to maintain rapid protein translation rate despite very low amino acid pools (Klumpp *et al*, 2013), there is no direct regulation of the peptide elongation rate in the regime of growth rates under consideration and so the translational efficiency γ will exhibit an unavoidable amino acid dependence. In the opposite limit, negative feedback regulation is in place to keep the amino acid pool from becoming too large (Neidhardt *et al*, 1990). Feedback regulation on amino acid transport is implemented by a variety of often overlapping mechanisms, including direct allosteric inhibition and combinatorial control of common transporters (Whipp & Pittard, 1977), and rho-dependent anti-termination (Quay & Oxender, 1977). Feedback regulation on biosynthesis is likewise implemented by a variety of molecular mechanisms including allosteric inhibition, transcriptional repression, attenuation, and covalent post-translational modification (Neidhardt *et al*, 1990). The overall effect of this regulation is to reduce the nutritional efficiency *ν* if the amino acid pool becomes too large. We now describe how these inefficient limiting cases (where flux is less than maximal) are avoided under favorable growth conditions.

To make explicit the general effect of the dependence of amino acid flux on the steady-state amino acid pool size a*, we model the translational efficiency 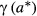 and nutritional efficiency 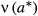 as simple sigmoidal functions,


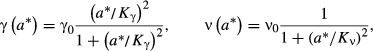
(15)

where translation becomes significantly attenuated for pool size a* below *K*_*γ*_, and the amino acid supply flux becomes significantly attenuated by feedback inhibition for a* above *K*_*ν*_. If the steady-state amino acid pool a* is kept between these two extremes, *K*_*γ*_ < a* < *K*_*ν*_, then the translational and nutritional efficiencies will be constant and close to maximal, 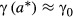 and 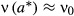, ensuring that the empirical linear relations [equations (1) and (2)] are recovered. The maximal efficiencies *γ*_0_ and *ν*_0_ are fixed by the environment; adding ribosome targeting antibiotics to the medium will lower *γ*_0_, whereas changes in nutrient composition of the growth medium will affect *ν*_0_. In this study, we will focus on growth changes due to changes in nutrient quality through *ν*_0_. We will assume that *ν*_0_ is fixed for a given growth medium composition and neglect adaptation to particular nutrients that occur on evolutionary timescales. Furthermore, we will hold *γ*_0_ constant, although it, too, can be modified by selective pressure on evolutionary timescales (Ehrenberg & Kurland, 1984; Okamoto & Savageau, 1984) (see also Supplementary Text S1).

Feedback inhibition in the amino acid supply flux via changes in the nutrient efficiency 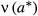 is one layer of regulation connecting amino acid flux balance and steady-state amino acid pool size a*; it is a classic end-product inhibition scheme (Savageau, 1975, 1977) that ensures the stability of the steady-state solution a* of the amino acid accumulation equation (12) for any admissible choice of the ribosomal protein fraction, 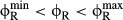. In reference to the schematic Fig 2A, irrespective of how the ribosomal protein fraction ϕ_R_ is set, if the amino acid pool increases beyond the level triggering feedback inhibition, *K*_*ν*_, then the nutritional efficiency 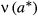 is reduced to keep the accumulation of the amino acid pool in check (much like a float-valve in a toilet tank). Although feedback inhibition is required for rapid adaptation to changes in the nutrient environment, as we show below, it plays a background role in the optimal regulation of ribosomal protein synthesis.

For a given ribosomal protein fraction ϕ_R_, flux balance then determines the steady-state amino acid pool a* via equations (6) and (13) (green line; Fig 2B),


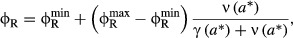
(16)

and, consequently, the growth rate


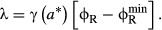
(17)

There is no unique choice of ribosomal protein fraction ϕ_R_ that will balance the flux; any pair 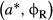 along the green line in Fig 2B will work. For the purpose of illustration, in Fig 2, the dynamic range of the steady-state amino acid pool size, *K*_*γ*_ < a* < *K*_*ν*_, is small so that the either the translational efficiency γ is reduced due to substrate limitation (green region; Fig 2B and C), or the nutritional efficiency is reduced due to feedback inhibition (pink region; Fig 2B and C) over most of the figure. Nevertheless, the growth rate [equation (17)] exhibits a unique maximum (green solid line; Fig 2C), defining the best choice for the ribosomal fraction ϕ_R_. The growth rate maximum attains a theoretical upper bound when the translation and amino acid supply rates are both maximal in a given growth environment at the steady state, 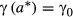 and 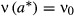 (closed circle, Fig 2C). We will refer to the theoretical upper bound as the “optimal” growth rate, denoted as 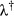, and denote the corresponding optimal ribosomal fraction as 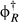; note that both 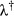 and 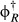 depend on the growth environment through *ν*_0_ (closed circles, Fig 2D).

### Regulatory strategies to attain optimal growth rate

We next investigate how the bacterium controls the ribosomal protein fraction ϕ_R_ in order to ensure that amino acid flux is balanced, and the regulatory strategies in place to bring this balance point as close to optimal as possible for a wide range of nutrient environments. We will show that these objectives can be realized simultaneously by keeping the dynamic range of the amino acid pool, *K*_*γ*_ < a* < *K*_*ν*_, as large as possible. In that case, supply-driven activation of ribosomal protein synthesis achieves amino acid flux balance and guarantees that growth rate will be maximal in different environments without any fine-tuning of the regulation.

## Control of ribosome synthesis

The balance of amino acid flux in exponential growth requires that the ribosomal protein fraction is set appropriately (Fig 2A and B). Here, we first review the molecular mechanisms that underlie the regulation of ribosomal protein synthesis. At its core, the regulation takes the amino acid pool size as a read-out of amino acid flux imbalance. By up-regulating ribosome synthesis when the amino acid pool size increases, flux balance can be achieved over a range of growth conditions.

The synthesis of ribosomal proteins is maintained by a subpopulation of the total active ribosomes. Molecularly, the synthesis of ribosomal proteins is controlled by the transcription of ribosomal RNA (rRNA) (Paul *et al*, 2004). The ribosomal proteins have strong affinity for binding to rRNA. When there is no rRNA to bind to, ribosomal proteins begin to accumulate in the cytoplasm and bind to their own mRNA to suppress its translation (Fallon *et al*, 1979). This post-transcriptional auto-regulation ensures that ribosomal protein translation is commensurate with ribosomal rRNA transcription, such that the control of ribosomal protein synthesis can be accomplished through the control of the synthesis of rRNA (Nomura *et al*, 1984). Synthesis of rRNA is, in turn, repressed by the alarmone ppGpp produced in response to uncharged tRNA (Condon *et al*, 1995; Murray *et al*, 2003; Potrykus *et al*, 2011). As a result, ppGpp mediates the increase in ribosomal protein synthesis when amino acids are abundant (high charged tRNA levels) and mediates the repression of ribosomal protein synthesis when amino acids are scarce (low charged tRNA levels).

**Figure 3:**
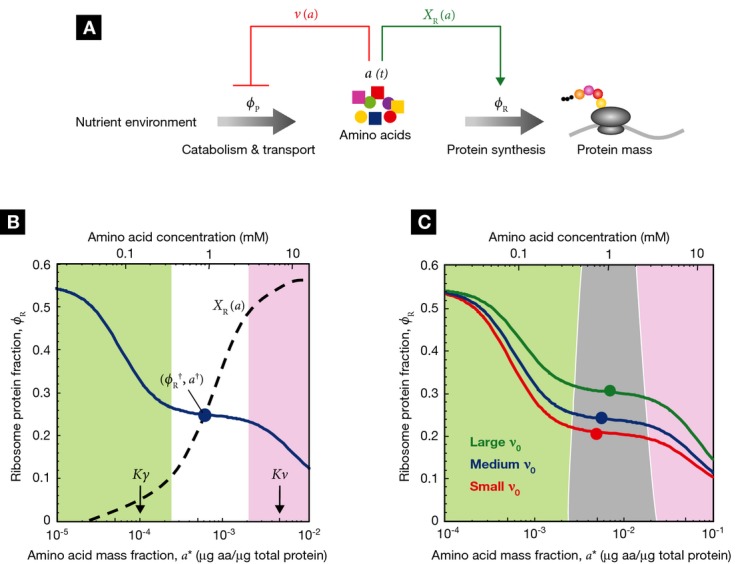
Regulation of the ribosomal protein fraction ϕ_R_ (A) Internal amino acid pools are kept in check by negative feedback inhibition 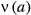 (red block arrow) via regulation of protein expression (described by *η*_*a*_) or allosteric inhibition (described by a decrease in efficacy *k*_*a*_). Negative feedback inhibition is important to rapidly regain steady-state growth upon nutrient shift, but plays an auxiliary role in growth rate maximization. When internal amino acid pools increase, supply-driven activation of ribosomal protein synthesis 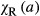 (green arrow) increases the rate of consumption to restore flux balance. (B) If the amino acid level for efficient elongation (*K*_*γ*_) and the level for negative feedback inhibition of amino acid supply (*K*_*ν*_) are well separated, *K*_*γ*_ << *K*_*ν*_, then the ribosomal protein fraction ϕ_R_ (blue solid line) is only weakly dependent on the steady-state amino acid level a* close to the optimal value 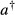 (filled circle) (lower axis displays amino acid level in units of mass fraction, upper axis displays the corresponding level in units of concentration). The intersection of 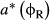 (blue line) and the ribosome synthesis function 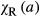 defines the steady state of the system (Supplementary Fig S2). A ribosome synthesis control function 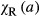 (dashed line) is shown passing through 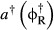 that yields the optimal ribosomal protein fraction 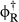 and growth rate 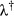. Notice that any curve intersecting ϕ_R_ in the plateau (white region) will return a steady-state ribosomal protein fraction close to the optimum, 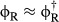. (C) Control functions 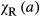 that pass through this plateau provide autonomous optimal control of the ribosomal protein fraction over a range of nutrient conditions. The dark gray band illustrates the range of control functions 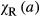 that determine ribosomal protein fraction ϕ_R_ to within 10% of the optimum 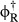 over a range of nutrient conditions. The colored lines and symbols correspond to those in Fig 2, with *ν*_0_ = 2.5/h (red), *ν*_0_ = 3.3/h (blue), and *ν*_0_ = 5.8/h (green); *K*_*γ*_ = 10^−4^, and *K*_*ν*_ = 50*K*_γ_ = 5 × 10^−3^. Experimental estimates for *K*_*γ*_, *K*_*ν*_, and steady-state amino acid pool sizes are given in Supplementary Table S1 (illustrated in Supplementary Fig S3).

Regulation of ribosomal protein synthesis by ppGpp is a feedforward scheme using tRNA charging as a measure of the imbalance between amino acid supply and consumption through protein synthesis. If amino acid pools increase, tRNA charging levels increase and ribosomal protein synthesis is de-repressed; in other words, increased supply flux activates ribosomal protein synthesis to restore flux balance in the system (green arrow; Fig 3A). We propose to call this feedforward regulatory motif “supply-driven activation”. It shares features with integral feedforward control (Leigh, 2004), insofar as the primary objective is flux balance irrespective of the steady-state amino acid concentration. Integral control has been implicated in the regulation of nitrogen uptake (Kim *et al*, 2012), and the coordination of nitrogen and carbon utilization (You *et al*, 2013); here, we suggest that a similar strategy coordinates amino acid supply and demand, resulting in optimal growth rate regulation over a range of nutrient conditions.

Let the fraction of ribosomes synthesizing ribosomal proteins be 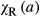, then the accumulation of total ribosomal protein mass *M*_R_ is [cf. equation (4)]


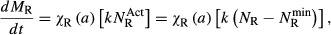
(18)

where 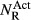 the number of active ribosomes. The fraction 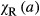 is determined by regulation that responds to the amino acid pool size *a*. In steady state, the amino acid pool size is a* and the ribosomal protein mass will accumulate exponentially, 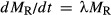. Dividing the equation for ribosomal protein synthesis, equation (18), by the total protein mass *M*,


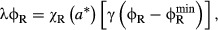
(19)

or, using equation (6),


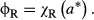
(20)

Thus, the control function 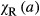 regulating the rate of ribosomal protein synthesis coincides, in steady state, with the mass fraction of ribosomal proteins ϕ_R_ and ultimately sets the growth rate via equation (6). In other words, if ribosomes represent 30% of the proteome in steady state, it also means that 30% of the ribosomes are engaged in producing ribosomes. In an unconstrained setting, the ribosomal protein fraction should be set as high as possible to maximize growth rate; however, the constraint imposed by the proteome partition results in an amino acid-limited translation rate if the ribosomal protein fraction is set too high.

The regulatory constraint equation (20), together with the amino acid flux balance constraint equation (16), uniquely determines the steady-state ribosomal protein fraction ϕ_R_ and the steady-state amino acid level a*. In the next section, we show that if the amino acid pool size has a large dynamic range between the limits where translation efficiency is reduced by inefficient tRNA charging and the limit where nutritional efficiency is reduced by feedback inhibition, then supply-driven activation of ribosomal protein synthesis implemented by an increasing regulatory function 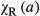 automatically achieves flux balance and the optimal growth rate for a given nutrient environment.

## Robust implementation of optimal regulation

When the amino acid pool is low, the system is self-limiting and amino acid consumption through protein synthesis decreases due to insufficient tRNA charging; in the opposite extreme, when the amino acid pool is high, negative feedback inhibition attenuates the supply flux. Between these two limits, both the translational and nutritional efficiencies are maximal for a given nutrient environment. Below, we show that not only does this produce a maximal flux through the system, but also facilitates regulation to achieve the optimal growth rate.

At first sight, it appears that the task of setting the steady-state ribosomal protein fraction ϕ_R_ to the optimum 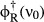 in a particular nutrient environment will require a fine-tuning of the ribosomal protein synthesis control function 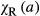. If, however, the amino acid level for efficient peptide elongation, *K*_*γ*_, is much less than the level for the negative feedback inhibition of amino acid supply, *K*_*ν*_ [cf. equation (15)], that is,


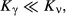
(21)

then for a range of amino acid levels


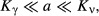
(22)

the rate of peptide elongation and amino acid supply will be maximal, 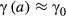, and 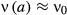, and negative feedback inhibition is not relevant. In the schematic Fig 2A, the large dynamic range corresponds to a deep reservoir for the amino acid pool. To better illustrate how condition [22] facilitates the location of a near-optimal steady-state solution 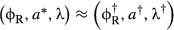, we provide a graphical illustration of the simultaneous solution of the flux balance constraint equation (16) and the regulatory constraint equation (20).

The flux balance constraint linking the ribosome–protein fraction ϕ_R_ and the steady-state amino acid level a* [equation (16)] is shown in Fig 3B as a solid blue line. The white vertical band indicates the region where the translation rate and amino acid supply rates are close to maximum, 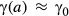, and 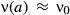, for a given growth environment. In this region, the ribosomal protein fraction ϕ_R_ is close to optimal and only weakly coupled to the amino acid level. We will refer to this white band as the “optimality plateau” in ϕ_R_. The larger the separation between the level for efficient peptide elongation *K*_*γ*_ and feedback regulation of amino acid supply *K*_*ν*_ [equation (21)], the wider this optimality plateau will be. In Supplementary Text S2, we show that the optimality plateau is further extended toward lower amino acids concentrations (i.e. below *K*_*γ*_) by co-regulation of the proteins involved in tRNA charging with ribosomal protein expression.

Graphically, the steady state, equation (20), corresponds to the intersection of 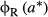 given by equation (16) (Fig 3B; blue line) and the ribosomal protein synthesis control function 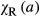 (Fig 3B; black dashed line). The control function 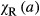 is shown passing through the optimum point 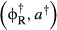 in Fig 3B (filled circle), although a function 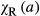 intersecting the blue line 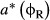 anywhere in the white optimality plateau yields a steady-state ribosomal protein fraction that is close to optimal 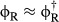,


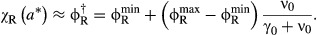
(23)

The optimality plateau in the ribosomal protein fraction ϕ_R_ is higher for better nutrient environment (larger *ν*_0_, green curve; Fig 3C), and lower for the opposite (smaller *ν*_0_, red curve; Fig 3C). To achieve optimum growth approximately, it is only necessary to have the control function 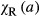 pass through the plateau region associated with each nutrient environment (closed circles; Fig 3C). Notice that without regulation, a constant fraction *χ*_R_ would intersect the optimality plateau for one particular value of the nutritional efficiency *ν*_0_; for all others, although amino acid flux would balance, the proteome partitioning would be non-optimal and the system would be operating under limitations in protein synthesis rate (green band; Fig 3C) or amino acid supply (pink band; Fig 3C). If ribosomal protein synthesis is regulated via supply-driven activation so that the control function 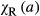 is an increasing function of the steady-state amino acid pool, then optimal growth rate is guaranteed irrespective of the nutrient environment (and the point of intersection is a stable global attractor for the system (Supplementary Fig S2)). The dark gray band in Fig 3C corresponds to the domain of the control function 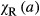 that determines the ribosomal protein fraction ϕ_R_ to within ± 10% of the optimum 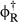. For a large dynamic range in the amino acid pool *K*_*γ*_ < < *K*_*ν*_, the optimality plateau is wide, and 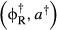 can be reached by a broad spectrum of putative control functions 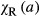 independent of the nutrient environment *ν*_0_.

It is important to notice that in the plateau region, where the translation rate and amino acid supply rates are constant and close to maximum, 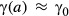, and 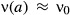, the ribosomal protein fraction ϕ_R_ is guaranteed to exhibit linear dependence on the growth rate λ through the constraint on protein mass accumulation equation, equation (17), and the constraint on amino acid flux balance, equation (13). As a consequence, a near-optimal ribosomal protein fraction 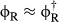 preserves the empirical linear correlation between ribosomal protein fraction and growth rate.

By incorporating the constraint imposed by proteome partitioning (Fig 1B) and the flux balance between protein synthesis and amino acid supply [equation (12)], the analysis suggests an automated control strategy to lock into optimal growth for a wide variety of nutrient environments *ν*_0_. If the amino acid level for efficient peptide elongation is well below the amino acid level for feedback inhibition of amino acid supply, *K*_*γ*_ << *K*_*ν*_, optimal growth rate can be achieved through supply-driven activation of ribosomal protein synthesis implemented via an increasing control function 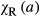. To investigate whether this strategy may be utilized by *E. coli* cells, we compare the values of *K*_*γ*_ and *K*_*ν*_ with the steady-state amino acid pools, a*.

The elongation rate 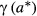 depends upon the intracellular amino acid abundance through binding of charged tRNA to the elongating ribosome. The available values of *K*_*γ*_ (taken to be the affinity of tRNA ligase for the cognate amino acid) are listed in Supplementary Table S1 (and displayed graphically by the green bars in Supplementary Fig S3), along with pool size estimates a* of a number of amino acids (blue bars in Supplementary Fig S3) (Maaløe, 1979; Bennett *et al*, 2009). [For the latter values, we used those pool sizes measured for *E. coli* grown in glucose minimal medium because the internal pools in medium supplemented with amino acids are extremely difficult to detect and are not available in the literature.] The data are, for the most part, consistent with the requirement that *K*_*γ*_ < a* (see also Hershey (1987)). The affinity for tRNA charging is of the order of 10–100 μM, while the amino acid pools are typically 10× to 100× larger. There are, however, several amino acids for which a* ≈ *K*_*γ*_ in exponential growth (e.g. trp, phe, tyr, met, thr, pro). These cases can be accommodated by the extension of the optimality plateau that results from co-regulation of proteins involved in tRNA charging with ribosomal proteins (see Supplementary Text S2).

Feedback regulation on amino acid transport and biosynthesis is implemented by a variety of often overlapping mechanisms, and negative feedback regulations occur on different nodes for different pathways depending upon the nature of the nutrients. Supplementary Table S1 lists some affinity estimates for allosteric inhibition and apo-repressor binding regulating amino acid biosynthesis (see the pink bars in Supplementary Fig S3 for a graphical display). These estimates do not include the elaborate complexity of some of the well-studied regulons [see for example van Heeswijk *et al* (2013)], or cases where negative feedback regulation may be implemented by a combination of signals (Lee *et al*, 1966; Woolfolk & Stadtman, 1967). Taking these tabulated values to be *K*_*ν*_ and comparing them to the amino acid pools a* (blue bars in Supplementary Fig S3), we see that for the most part, a* < *K*_*ν*_. A few amino acids are larger than their respective inhibitory constants and are unlikely to be the growth-limiting amino acids (e.g. glutamate, with its extraordinarily large pool). Since the violation of the condition a* < *K*_*ν*_ would imply the setting of the cell's regulatory mechanisms to significantly impede its growth (because 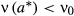 in this case), we assert this condition as a biologically reasonable conjecture for the growth-limiting amino acids. There is a well-known counter-example to this conjecture, which is the growth arrest of certain *E. coli* strains in minimal media upon the addition of valine. This is, however, due to broken regulation in these domesticated strains (De Felice *et al*, 1979), and is unlikely to occur in growth conditions commonly encountered by wild-type bacteria.

## Discussion

Despite the complexity of molecular networks, biological organisms display remarkably robust properties at higher levels of organization, prompting conjectures on the modularity of biological organizations (Hartwell *et al*, 1999; Arkin & Fletcher, 2006; Guido *et al*, 2006). Naively, one might expect modularity of cellular organization to insulate typical genetic circuits from the physiology of cell growth. This does not appear to be the case (Klumpp *et al*, 2009); nevertheless, the interactions between cell growth and gene expression follow surprisingly simple rules in bacteria (Scott *et al*, 2010; You *et al*, 2013). A prototypical example is the linear dependence of the ribosomal content on the growth rate under changes in nutrient quality or translational efficiency, that is, the growth laws (Fig 1A). In this work, we have addressed the molecular origin of these growth laws.

In balanced exponential growth, the maximal growth rate is ultimately determined by the rate at which nutrients in the environment can be converted to protein mass. Focusing on the flux of amino acids, the growth rate is maximal if both the rate of supply and consumption are maximal. A key challenge the bacterium faces is how to maintain maximal growth rate over a wide variety of nutrient environments. Neglecting genetic change on evolutionary timescales (see Supplementary Text S1), we identify two interlocking regulatory loops that provide automated coordination of the amino acid flux between supply and consumption—the first is a classical end-product inhibition of amino acid supply and the second is supply-driven activation of ribosomal protein synthesis. Below, we explore the biological significance of the analysis in more detail.

### Balance of supply and demand

One of the most basic transaction in the “economy” of cell growth is the conversion of environmental nutrients to protein biomass (Molenaar *et al*, 2009), with amino acid abundance acting as the common currency linking metabolism with protein synthesis. Consumption of amino acids by protein synthesis is limited at low amino acid levels by physical–chemical constraints on tRNA charging. There are several mechanisms the cell employs to keep that limit as low as possible; tRNA charging is efficient, and the absolute abundance of tRNA-affiliated proteins is kept high ensuring that the ribosome is nearly saturated with charged tRNA down to very low amino acid levels (see, for example, Klumpp *et al*, 2013). The effective amino acid supply rate is typically regulated by end-product inhibition, a prevalent motif in metabolic regulation whereby an increase in the level of a product inhibits a preceding step in the pathway. This type of regulation offers many advantages if the objective is to keep a fixed product concentration (Savageau, 1975), although, as discussed below, product homeostasis is not the primary objective in managing amino acid flux.

**Figure 4:**
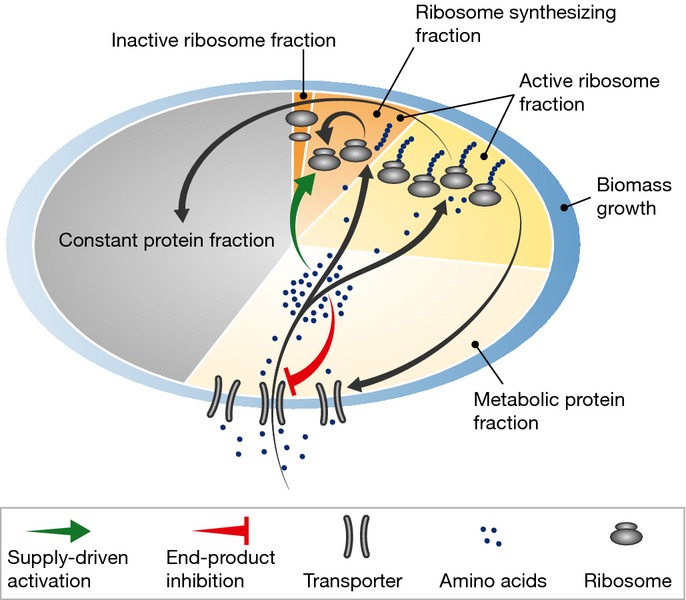
Schematic illustration of the growth model The analysis identifies amino acid flux as a primary transaction during exponential growth, with supply rate proportional to the metabolic protein fraction and consumption through protein synthesis. If the amino acid pool becomes too large, negative feedback regulation attenuates the supply flux (red block arrow) and guarantees the system can reach a stable equilibrium. Supply-driven activation of ribosomal protein synthesis ensures optimal allocation of cellular resources by monitoring amino acid incorporation at the ribosome (green arrow)—the regulation is agnostic about the details of the supply. As a result, there is an inherent plasticity in the system. Specific catabolic pathways can be turned on and off depending upon the nutrient environment, with regulation of ribosomal protein synthesis automatically adjusting the rate of amino acid consumption to optimize growth rate. From an evolutionary perspective, the coarse-grained modularity in the system, with demand flux adjusted to the supply, allows innovative metabolic proteins and pathways to be swapped into the genome with robust regulation of ribosome synthesis ensuring maximal growth rate.

At a coarse-grained level, the proteome can be partitioned into different fractions, including a ribosomal protein fraction and a metabolic protein fraction (Fig 4). There is a natural constraint that emerges from this partitioning: if the fraction of ribosomal protein increases, then the fraction of metabolic protein must necessarily decrease.

For any choice of the ribosomal protein fraction, there is an amino acid pool size that balances the supply and demand (Fig 2A and B)—the challenge faced by the organism is not only the balance of supply and demand, but how to choose the ribosomal protein fraction that maximizes amino acid flux (e.g. by avoiding too high an amino acid pool which would have reduced amino acid supply flux), and thereby maximizing the growth rate.

Mechanistically, regulation of ribosomal protein synthesis is tied to the amino acid pool size: Increased supply flux of amino acids activates ribosomal protein synthesis to restore flux balance in the system (green arrow; Fig 4). We call this feedforward regulatory motif “supply-driven activation”. Supply-driven activation (and its dynamic counterpart, integral feedforward regulation) is used to balance flux among other modular networks such as nitrogen uptake (Kim *et al*, 2012), the metabolic regulation of glycolysis (Kochanowski *et al*, 2012), and carbon and nitrogen utilization (You *et al*, 2013). It may be that this strategy can be used to coordinate flux between endogenous and synthetic pathways in engineered organisms. More generally, one could imagine that assembly along these lines, with large networks from diverse organisms stitched together and held in place by supply-driven activation, offers a promising approach to the design of whole synthetic organisms.

### Origin of robust regulation

More than 60 years ago, Monod drew attention to the simple laws that emerge from bacterial growth “despite the immense complexity of the phenomena to which it testifies” (Monod, 1949). Phenomenological studies of bacterial growth and growth rate regulation have been used throughout the intervening decades to great success (for a review, see Scott & Hwa, 2011), and one of their chief advantages is that strong predictive models can be built in the absence of information about molecular details. It is important to emphasize, however, that the independence of phenomenological approaches from molecular mechanisms does not imply that molecular controls are not important to the phenomena. On the contrary, empirical laws in biology may generally suggest the existence of underlying robust regulatory strategies and pinpoint their molecular implementations, as demonstrated recently for the problem of cAMP signaling that has evaded 50 years of genetic and high-throughput studies (You *et al*, 2013).

In this work, we have used empirical constraints linking ribosome abundance and growth rate [equations (1) and (2); Fig 1A] to identify a feature of ribosome synthesis regulation that renders the system insensitive to the specific details of the regulatory function. A separation between the internal amino acid level required for efficient peptide elongation and level required to initiate negative feedback inhibition of amino acid supply [equation (18)] results in an “optimality plateau”; rather than fine-tuning to hit a bull's eye, the challenge of optimal regulation shifts to hitting a much broader objective. De-repression of ribosomal protein synthesis by ppGpp in response to amino acid accumulation is enough to guarantee the optimum is achieved in any nutrient environment irrespective of the detailed amino acid dependence of the de-repression.

A remarkable feature of the control strategies underlying optimal growth rate regulation is that they maintain a linear relationship between macroscopic variables (e.g. ribosome concentration and growth rate), despite the highly nonlinear biochemical reaction networks that drive cell growth. In the optimality plateau, the amino acid supply rate and the protein elongation rate are both close to maximal and are determined by the composition of the growth medium, irrespective of the intracellular amino acid abundance.

A major direction in systems biology is to identify and understand the emerging robustness of biological systems from unreliable molecular components (Hartwell *et al*, 1999; Guido *et al*, 2006). It is our belief that quantitative empirical characterization may lead to the discovery of additional phenomenological laws that, in turn, reveal global constraints and robust regulatory strategies that give rise to the reliable performance of living systems. Although our focus is on exponential growth, the existence of a growth rate maximum is only part of the picture; the dynamics of how the system approaches the optimum is equally important. A phenomenological approach applied to the dynamics of adaptation reveals additional constraints on regulation and resource allocation (Pavlov & Ehrenberg, 2014). A coupling of dynamic proteome partitioning with ribosome synthesis promises to provide a more complete picture of how global regulation is used to couple physiology to changes in the growth environment.

### Phenomenological models and coarse-grained modular design

Mathematical studies of biomolecular systems are dominated by the “bottom-up” approach, that is, starting with known molecular features and including mutual interactions to predict system-level properties (Guido *et al*, 2006). While the bottom-up approach has been successful in analyzing small-scale systems where most of the interactions have been characterized, it becomes increasingly difficult to implement as one moves toward larger systems, where the number of parameters “explodes” (Kwok, 2010). In the present study involving cell growth, a complete bottom-up approach is impossible due to the lack of knowledge of many processes regulating growth. In fact, the bottom-up approach cannot even predict the growth rate dependence of gene expression from an unregulated (or “constitutive”) promoter, which is often taken as the reference state in the study of gene regulatory processes [see (Klumpp *et al*, 2009) for more discussion of the shortcomings of the bottom-up approach]. Existing computational models of cell growth (Tadmor & Tlusty, 2008; Bollenbach *et al*, 2009; Karr *et al*, 2012; Labhsetwar *et al*, 2013) have adopted varying degrees of coarse graining of the growth process and taken advantage of the extraordinarily meticulous characterization of the growth physiology of *E. coli* (Neidhardt *et al*, 1987; Bremer & Dennis, 1996; Scott & Hwa, 2011). It would be a considerable task to generate such detailed information for other organisms, or even for *E. coli* subjected to different modes of growth limitation.

In contrast, the theory presented here requires a minimum of molecular level information. It is based upon a “top-down”, or phenomenological, approach that relies on empirical growth laws, constraints on protein allocation, and the known topology of regulatory interactions. Identifying amino acid flux as a dominant contribution to bacterial growth rate and growth rate regulation, the schematic picture that emerges is an extension of the program begun by Maaløe many decades ago (Maaløe, 1979), with the addition of a metabolic protein fraction responsible for amino acid supply (Fig 4).

A coarse-grained view of bacterial growth makes clear the intrinsic plasticity in the regulation of metabolism. Regulation of ribosome synthesis directly controls amino acid demand flux via ppGpp and indirectly controls supply flux via the proteome partitioning constraint (Fig 1B)—if amino acid levels rise in the cell, then the mass fraction of ribosomal proteins is increased to restore amino acid flux balance, and simultaneously, the mass fraction of metabolic proteins is decreased to attenuate the supply flux. The supply-driven feedforward regulation is directed toward the synthesis of ribosomal protein (green arrow; Fig 4) and operates independently of whatever sensing, transport or metabolic processing the organism requires to generate an influx of amino acids. Consequently, at a coarse-grained level, the system is partitioned into autonomous “modules” of metabolism and protein synthesis, with metabolism acting as a “black-box” amino acid source [see also (Kotte *et al*, 2010)]. Evidence of this coarse-grained modularity comes from the observed linear relation between ribosomal proteins and growth rate [equation (1)] despite substantial changes to the nutrient content of the growth media (Scott *et al*, 2010). Although the identity of the “growth-limiting” amino acid may change with the nutrient conditions, supply-driven activation of ribosomal protein synthesis by ppGpp is sensitive only to flux mismatch, and consequently, ppGpp levels exhibit negative correlation with growth rate irrespective of the composition of the growth medium (Potrykus *et al*, 2011).

From a broader perspective, coarse-grained modularity could facilitate bacterial diversification. Comparative genomic studies have identified a core list of about 500 persistent genes conserved in a large number of bacteria (called the “paleome”) and thousands of non-persistent genes that allow growth in niche environments (called the “cenome”, or “community genome”) (Acevedo-Rocha *et al*, 2012). The same plasticity that allows autonomous metabolic regulation to interface with protein synthesis would likewise accommodate the evolution of innovative metabolic genes and networks acquired by horizontal transfer from the community cenome. Here, we have an example of primordial plug-and-play; a self-configuring system poised to cannibalize from surrounding organisms viable solutions to local problems of sensing, adaptation and nutrient processing.

## References

[b1] Acevedo-Rocha CG, Fang G, Schmidt M, Ussery DW, Danchin A (2012). From essential to persistent genes: a functional approach to constructing synthetic
life. Trends Genet.

[b2] Arkin AP, Fletcher DA (2006). Fast, cheap and somewhat in control. Genome Biol.

[b3] Armstrong JB, Fairfield JA (1975). A new method for the isolation of methionyl transfer RNA synthetase mutants from
*Escherichia coli*. Can J Microbiol.

[b4] Ataide SF, Ibba M (2004). Discrimination of cognate and noncognate substrates at the active site of class II
lysyl-tRNA synthetase. Biochemistry.

[b5] Bennett BD, Kimball EH, Gao M, Osterhout R, Van Dien SJ, Rabinowitz JD (2009). Absolute metabolite concentrations and implied enzyme active site occupancy in
*Escherichia coli*. Nat Chem Biol.

[b6] Bollenbach T, Quan S, Chait R, Kishony R (2009). Nonoptimal microbial response to antibiotics underlies suppressive drug
interactions. Cell.

[b7] Borel F, Vincent C, Leberman R, Hartlein M (1994). Seryl-tRNA synthetase from *Escherichia coli*: implication of its
N-terminal domain in aminoacylation activity and specificity. Nucleic Acids Res.

[b8] Bremer H, Dennis PP, Neidhardt FC (1996). Modulation of chemical composition and other parameters of the cell by growth
rate. Escherichia coli and Salmonella.

[b9] Cedar H, Schwartz JH (1969). The asparagine synthetase of *Escherichia coli*. II. Studies on
mechanism. J Biol Chem.

[b10] Condon C, Squires C, Squires CL (1995). Control of rRNA transcription in *Escherichia coli*. Microbiol Rev.

[b11] Cooper S (1993). The origins and meaning of the Schaechter-Maaloe-Kjeldgaard
experiments. J Gen Microbiol.

[b12] De Felice M, Levinthal M, Iaccarino M, Guardiola J (1979). Growth inhibition as a consequence of antagonism between related amino acids: effect
of valine in *Escherichia coli* K-12. Microbiol Rev.

[b13] Deris JB, Kim M, Zhang Z, Okano H, Hermsen R, Groisman A, Hwa T (2013). The innate growth bistability and fitness landscapes of antibiotic-resistant
bacteria. Science.

[b14] Dopheide TA, Crewther P, Davidson BE (1972). Chorismate mutase-prephenate dehydratase from *Escherichia coli*
K-12. J Biol Chem.

[b15] Ehrenberg M, Kurland CG (1984). Costs of accuracy determined by a maximal growth rate constraint. Q Rev Biophys.

[b16] Elf J, Ehrenberg M (2005). Near-critical behavior of aminoacyl-tRNA pools in *Escherichia coli*
at rate-limiting supply of amino acids. Biophys J.

[b17] Eriani G, Dirheimer G, Gangloff J (1990). Structure-function relationship of arginyl-tRNA synthetase from *Escherichia
coli*: isolation and characterization of the argS mutation MA5002. Nucleic Acids Res.

[b18] Fallon AM, Jinks CS, Strycharz GD, Nomura M (1979). Regulation of ribosomal protein synthesis in *Escherichia coli* by
selective mRNA inactivation. Proc Natl Acad Sci USA.

[b19] Filley SJ, Hill KA (1993). Amino acid substitutions at position 73 in motif 2 of *Escherichia
coli* alanyl-tRNA synthetase. Arch Biochem Biophys.

[b20] Geng F, Chen Z, Zheng P, Sun J, Zeng AP (2013). Exploring the allosteric mechanism of dihydrodipicolinate synthase by reverse
engineering of the allosteric inhibitor binding sites and its application for lysine
production. Appl Microbiol Biotechnol.

[b21] Guido NJ, Wang X, Adalsteinsson D, McMillen D, Hasty J, Cantor CR, Elston TC, Collins JJ (2006). A bottom-up approach to gene regulation. Nature.

[b22] Hartwell LH, Hopfield JJ, Leibler S, Murray AW (1999). From molecular to modular cell biology. Nature.

[b23] van Heeswijk WC, Westerhoff HV, Boogerd FC (2013). Nitrogen assimilation in *Escherichia coli*: putting molecular data
into a systems perspective. Microbiol Mol Biol Rev.

[b24] Herring CD, Raghunathan A, Honisch C, Patel T, Applebee MK, Joyce AR, Albert TJ, Blattner FR, van den Boom D, Cantor CR, Palsson BO (2006). Comparative genome sequencing of *Escherichia coli* allows observation
of bacterial evolution on a laboratory timescale. Nat Genet.

[b25] Hershey JWB, Neidhardt FC, Ingraham JL, Brooks KL, Magasanik B, Schaechter M, Umbarger HE (1987). Protein synthesis. Escherichia coli and Salmonella typhimurium: Cellular and Molecular Biology.

[b26] Howe JG, Hershey JW (1983). Initiation factor and ribosome levels are coordinately controlled in
*Escherichia coli* growing at different rates. J Biol Chem.

[b27] James CL, Viola RE (2002). Production and characterization of bifunctional enzymes. Domain swapping to produce
new bifunctional enzymes in the aspartate pathway. Biochemistry.

[b28] Jin L, Xue WF, Fukayama JW, Yetter J, Pickering M, Carey J (2005). Asymmetric allosteric activation of the symmetric ArgR hexamer. J Mol Biol.

[b29] Karr JR, Sanghvi JC, Macklin DN, Gutschow MV, Jacobs JM, Bolival B, Assad-Garcia N, Glass JI, Covert MW (2012). A whole-cell computational model predicts phenotype from genotype. Cell.

[b30] Kern D, Potier S, Lapointe J, Boulanger Y (1980). The glutaminyl-transfer RNA synthetase of *Escherichia coli*.
Purification, structure and function relationship. Biochim Biophys Acta.

[b31] Keseler IM, Mackie A, Peralta-Gil M, Santos-Zavaleta A, Gama-Castro S, Bonavides-Martinez C, Fulcher C, Huerta AM, Kothari A, Krummenacker M, Latendresse M, Muniz-Rascado L, Ong Q, Paley S, Schroder I, Shearer AG, Subhraveti P, Travers M, Weerasinghe D, Weiss V (2013). EcoCyc: fusing model organism databases with systems biology. Nucleic Acids Res.

[b32] Kiga D, Sakamoto K, Kodama K, Kigawa T, Matsuda T, Yabuki T, Shirouzu M, Harada Y, Nakayama H, Takio K, Hasegawa Y, Endo Y, Hirao I, Yokoyama S (2002). An engineered *Escherichia coli* tyrosyl-tRNA synthetase for
site-specific incorporation of an unnatural amino acid into proteins in eukaryotic translation and
its application in a wheat germ cell-free system. Proc Natl Acad Sci USA.

[b33] Kim M, Zhang Z, Okano H, Yan D, Groisman A, Hwa T (2012). Need-based activation of ammonium uptake in *Escherichia
coli*. Mol Syst Biol.

[b34] Klumpp S, Zhang Z, Hwa T (2009). Growth rate-dependent global effects on gene expression in bacteria. Cell.

[b35] Klumpp S, Scott M, Pedersen S, Hwa T (2013). Molecular crowding limits translation and cell growth. Proc Natl Acad Sci USA.

[b36] Klumpp S, Hwa T (2014). Bacterial growth: global effects on gene expression, growth feedback and proteome
partition. Curr Opin Biotechnol.

[b37] Kochanowski K, Volkmer B, Gerosa L, Haverkorn van Rijsewijk BR, Schmidt A, Heinemann M (2012). Functioning of a metabolic flux sensor in *Escherichia
coli*. Proc Natl Acad Sci USA.

[b38] Kotte O, Zaugg JB, Heinemann M (2010). Bacterial adaptation through distributed sensing of metabolic fluxes. Mol Syst Biol.

[b39] Kubitschek HE, Baldwin WW, Schroeter SJ, Graetzer R (1984). Independence of buoyant cell density and growth rate in *Escherichia
coli*. J Bacteriol.

[b40] Kwok R (2010). Five hard truths for synthetic biology. Nature.

[b41] Labhsetwar P, Cole JA, Roberts E, Price ND, Luthey-Schulten ZA (2013). Heterogeneity in protein expression induces metabolic variability in a modeled
*Escherichia coli* population. Proc Natl Acad Sci USA.

[b42] Lee LW, Ravel JM, Shive W (1966). Multimetabolite control of a biosynthetic pathway by sequential
metabolites. J Biol Chem.

[b43] Leigh JR (2004). Control Theory.

[b44] Li GW, Burkhardt D, Gross C, Weissman JS (2014). Quantifying absolute protein synthesis rates reveals principles underlying allocation
of cellular resources. Cell.

[b45] Lue SW, Kelley SO (2005). An aminoacyl-tRNA synthetase with a defunct editing site. Biochemistry.

[b46] Maaløe O, Goldberger RF (1979). Regulation of the protein-synthesizing machinery – ribosomes, tRNA, factors,
and so on. Biological Regulation and Development.

[b47] Madern D, Anselme J, Hartlein M (1992). Asparaginyl-tRNA synthetase from the *Escherichia coli*
temperature-sensitive strain HO202. A proline replacement in motif 2 is responsible for a large
increase in Km for asparagine and ATP. FEBS Lett.

[b48] Martin F, Sharples GJ, Lloyd RG, Eiler S, Moras D, Gangloff J, Eriani G (1997). Characterization of a thermosensitive *Escherichia coli* aspartyl-tRNA
synthetase mutant. J Bacteriol.

[b49] Miller RE, Stadtman ER (1972). Glutamate synthase from *Escherichia coli*. An iron-sulfide
flavoprotein. J Biol Chem.

[b50] Milo R (2013). What is the total number of protein molecules per cell volume? A call to rethink some
published values. BioEssays.

[b51] Molenaar D, van Berlo R, de Ridder D, Teusink B (2009). Shifts in growth strategies reflect tradeoffs in cellular economics. Mol Syst Biol.

[b52] Monod J (1949). The growth of bacterial cultures. Ann Rev Microbiol.

[b53] Murray HD, Schneider DA, Gourse RL (2003). Control of rRNA expression by small molecules is dynamic and
nonredundant. Mol Cell.

[b54] Neidhardt FC, Ingraham JL, Magasanik B, Low KB, Schaechter M, Umbarger HE (1987). Escherichia coli and Salmonella typhimurium.

[b55] Neidhardt FC, Ingraham JL, Schaechter M (1990). Physiology of the Bacterial Cell: A Molecular Approach.

[b56] Neidhardt FC, Bloch PL, Pedersen S, Reeh S (1977). Chemical measurement of steady-state levels of ten aminoacyl-transfer ribonucleic
acid synthetases in *Escherichia coli*. J Bacteriol.

[b57] Nierhaus KH, Nierhaus KH, Wilson DN (2004). The elongation cycle. Protein Synthesis and Ribosome Structure.

[b58] Nomura M, Gourse R, Baughman G (1984). Regulation of the synthesis of ribosomes and ribosomal components. Annu Rev Biochem.

[b59] Okamoto M, Savageau MA (1984). Integrated function of a kinetic proofreading mechanism: dynamic analysis separating
the effects of speed and substrate competition on accuracy. Biochemistry.

[b60] Paul BJ, Ross W, Gaal T, Gourse RL (2004). rRNA transcription in *Escherichia coli*. Annu Rev Genet.

[b61] Pavlov MY, Freistroffer DV, MacDougall J, Buckingham RH, Ehrenberg M (1997). Fast recycling of *Escherichia coli* ribosomes requires both ribosome
recycling factor (RRF) and release factor RF3. EMBO J.

[b62] Pavlov MY, Ehrenberg M (2014). Optimal control of gene expression for fast proteome adaptation to environmental
change. Proc Natl Acad Sci USA.

[b63] Pedersen S, Bloch PL, Reeh S, Neidhardt FC (1978). Patterns of protein synthesis in *Escherichia coli*: a catalog of the
amount of 140 individual proteins at different growth rates. Cell.

[b64] Potrykus K, Murphy H, Philippe N, Cashel M (2011). ppGpp is the major source of growth rate control in *Escherichia
coli*. Environ Microbiol.

[b65] Quay SC, Oxender DL (1977). Regulation of amino acid transport in *Escherichia coli* by
transcription termination factor rho. J Bacteriol.

[b66] Rodnina MV, Gromadski KB, Kothe U, Wieden HJ (2005). Recognition and selection of tRNA in translation. FEBS Lett.

[b67] Roy H, Ling J, Irnov M, Ibba M (2004). Post-transfer editing in vitro and in vivo by the beta subunit of phenylalanyl-tRNA
synthetase. EMBO J.

[b68] Ruhlmann A, Cramer F, Englisch U (1997). Isolation and analysis of mutated histidyl-tRNA synthetases from *Escherichia
coli*. Biochem Biophys Res Commun.

[b69] Sankaranarayanan R, Dock-Bregeon AC, Rees B, Bovee M, Caillet J, Romby P, Francklyn CS, Moras D (2000). Zinc ion mediated amino acid discrimination by threonyl-tRNA
synthetase. Nat Struct Biol.

[b70] Savageau MA (1975). Optimal design of feedback control by inhibition: dynamic
considerations. J Mol Evol.

[b71] Savageau MA (1977). Biochemical Systems Analysis: Study of Function and Design in Molecular Biology.

[b72] Schaechter M, Maaloe O, Kjeldgaard NO (1958). Dependency on medium and temperature of cell size and chemical composition during
balanced grown of Salmonella typhimurium. J Gen Microbiol.

[b73] Schomburg I, Chang A, Hofmann O, Ebeling C, Ehrentreich F, Schomburg D (2002). BRENDA: a resource for enzyme data and metabolic information. Trends Biochem Sci.

[b74] Scott M, Gunderson CW, Mateescu EM, Zhang Z, Hwa T (2010). Interdependence of cell growth and gene expression: origins and
consequences. Science.

[b75] Scott M, Hwa T (2011). Bacterial growth laws and their applications. Curr Opin Biotechnol.

[b76] Sekine S, Nureki O, Tateno M, Yokoyama S (1999). The identity determinants required for the discrimination between tRNAGlu and tRNAAsp
by glutamyl-tRNA synthetase from *Escherichia coli*. Eur J Biochem.

[b77] Sorensen MA (2001). Charging levels of four tRNA species in *Escherichia coli* Rel+
and Rel− strains during amino acid starvation: a simple model for the effect of ppGpp on
translational accuracy. J Mol Biol.

[b78] Stadtman ER, Cohen GN, Lebras G (1961). Feedback inhibition and repression of aspartokinase activity in *Escherichia
coli*. Ann N Y Acad Sci.

[b79] Stehlin C, Heacock DH, Liu H, Musier-Forsyth K (1997). Chemical modification and site-directed mutagenesis of the single cysteine in motif 3
of class II *Escherichia coli* prolyl-tRNA synthetase. Biochemistry.

[b80] Sterboul CC, Kleeman JE, Parsons SM (1977). Purification and characterization of a mutant ATP phosphoribosyltransferase
hypersensitive to histidine feedback inhibition. Arch Biochem Biophys.

[b81] Sugimoto E, Pizer LI (1968). The mechanism of end product inhibition of serine biosynthesis. I. Purification and
kinetics of phosphoglycerate dehydrogenase. J Biol Chem.

[b82] Tadmor AD, Tlusty T (2008). A coarse-grained biophysical model of *Escherichia coli* and its
application to perturbation of the rRNA operon copy number. PLoS Comput Biol.

[b83] Tardif KD, Horowitz J (2004). Functional group recognition at the aminoacylation and editing sites of
*Escherichia coli* valyl-tRNA synthetase. RNA.

[b84] Turnbull J, Morrison JF, Cleland WW (1991). Kinetic studies on chorismate mutase-prephenate dehydrogenase from
*Escherichia coli*: models for the feedback inhibition of prephenate dehydrogenase by
L-tyrosine. Biochemistry.

[b85] Whipp MJ, Pittard AJ (1977). Regulation of aromatic amino acid transport systems in *Escherichia
coli* K-12. J Bacteriol.

[b86] White D, Drummond J, Fuqua C (2011). The Physiology and Biochemistry of Prokaryotes.

[b87] Wintermeyer W, Peske F, Beringer M, Gromadski KB, Savelsbergh A, Rodnina MV (2004). Mechanisms of elongation on the ribosome: dynamics of a macromolecular
machine. Biochem Soc Trans.

[b88] Woolfolk CA, Stadtman ER (1967). Regulation of glutamine synthetase. 3. Cumulative feedback inhibition of glutamine
synthetase from *Escherichia coli*. Arch Biochem Biophys.

[b89] You C, Okano H, Hui S, Zhang Z, Kim M, Gunderson CW, Wang YP, Lenz P, Yan D, Hwa T (2013). Coordination of bacterial proteome with metabolism by cyclic AMP
signalling. Nature.

[b90] Zaslaver A, Kaplan S, Bren A, Jinich A, Mayo A, Dekel E, Alon U, Itzkovitz S (2009). Invariant distribution of promoter activities in *Escherichia
coli*. PLoS Comput Biol.

[b91] Zuniga R, Salazar J, Canales M, Orellana O (2002). A dispensable peptide from Acidithiobacillus ferrooxidans tryptophanyl-tRNA
synthetase affects tRNA binding. FEBS Lett.

