# Supplementary figures and images for: Emergence of robust growth laws from optimal regulation of ribosome synthesis
Source: Mol Syst Biol. 2014 Aug 22;10(8):747. doi: 10.15252/msb.20145379 (PMC4299513; doi:10.15252/msb.20145379)

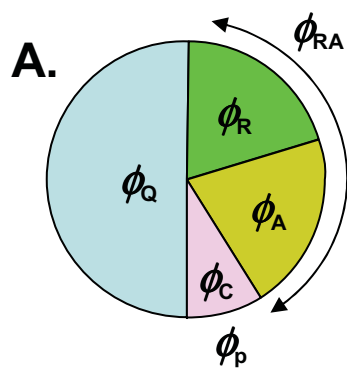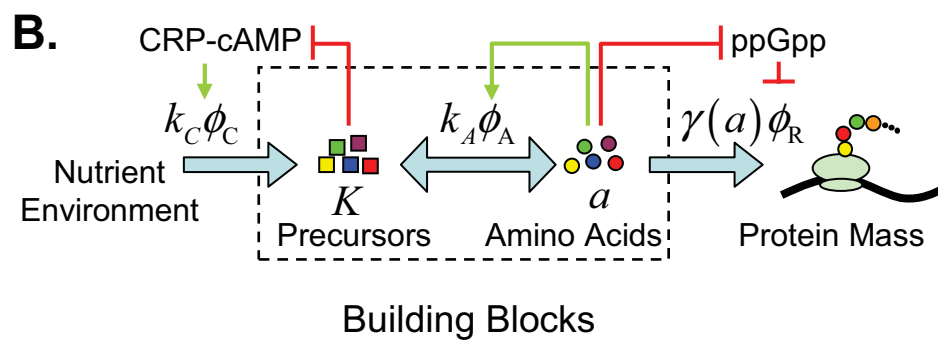

Supplement: Supplementary file 1 — Supplementary Figure S1 [file msb0010-0747-SD1.pdf]

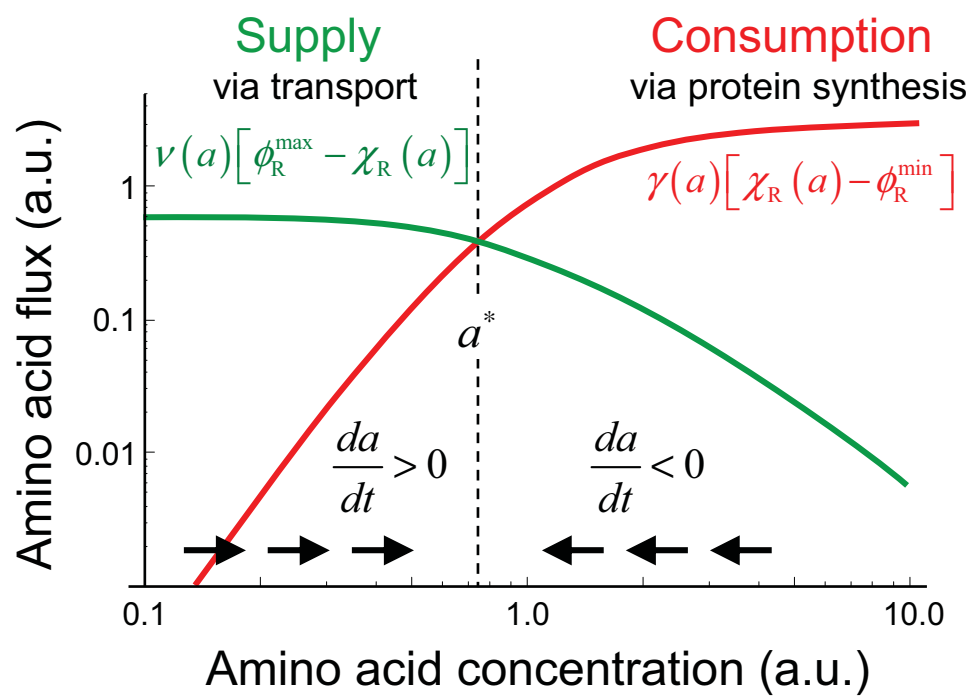

Supplement: Supplementary file 2 — Supplementary Figure S2 [file msb0010-0747-SD2.pdf]

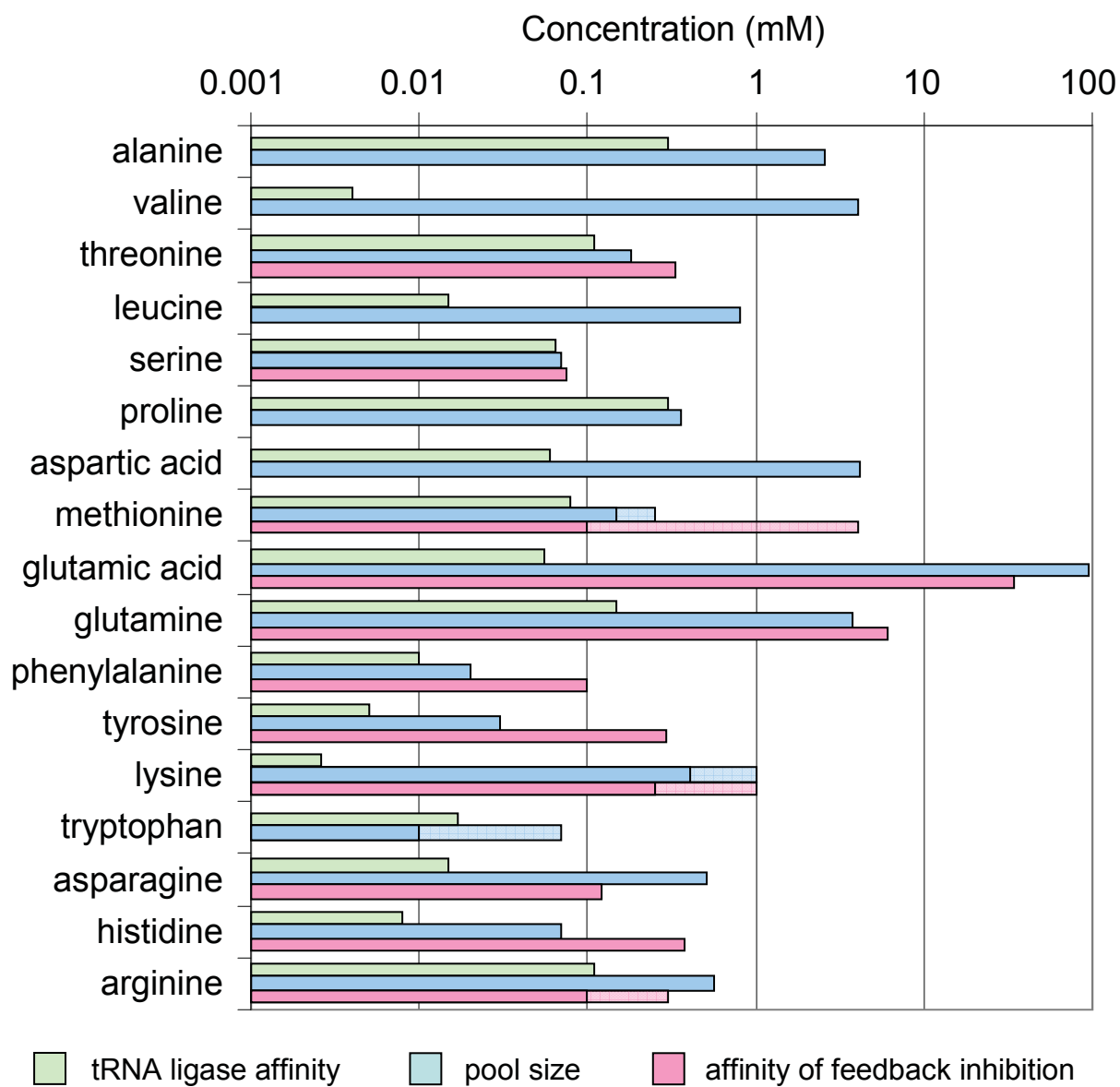

Supplement: Supplementary file 3 — Supplementary Figure S3 [file msb0010-0747-SD3.pdf]

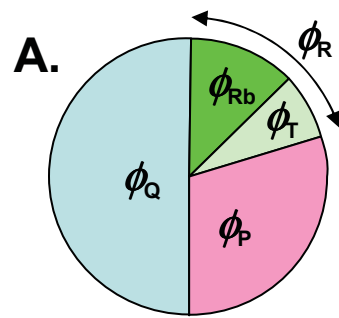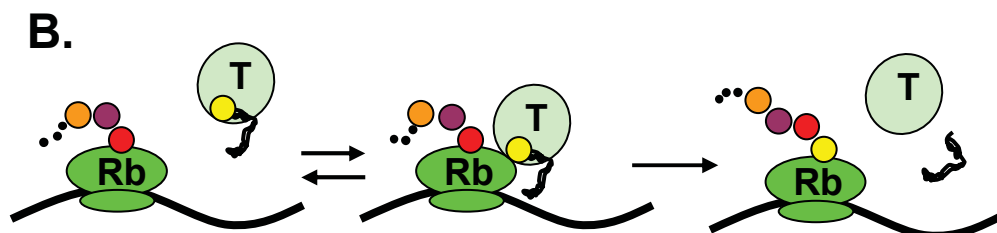

Supplement: Supplementary file 4 — Supplementary Figure S4 [file msb0010-0747-SD4.pdf]

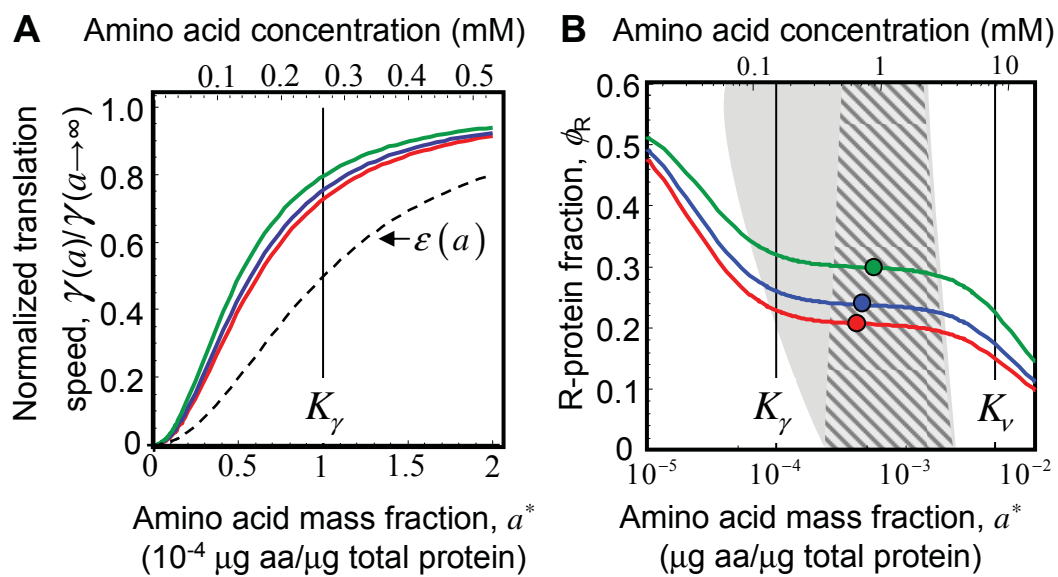

Supplement: Supplementary file 5 — Supplementary Figure S5 [file msb0010-0747-SD5.pdf]

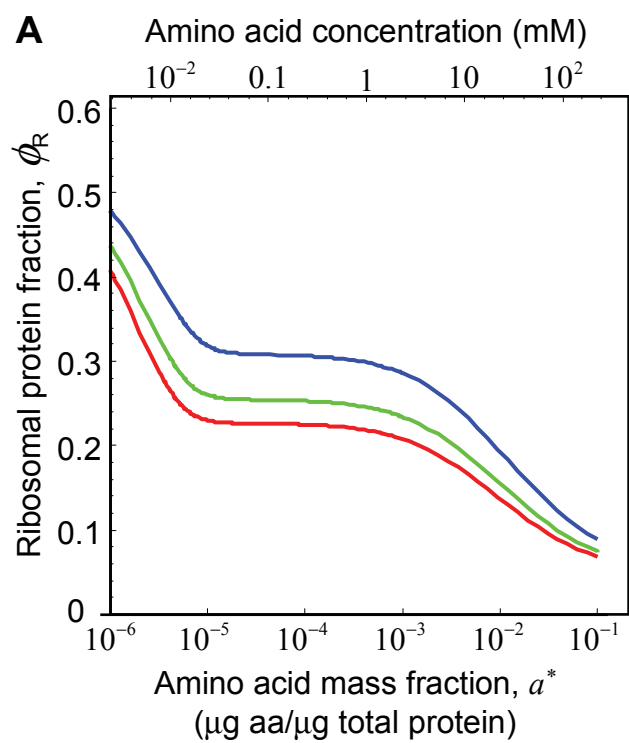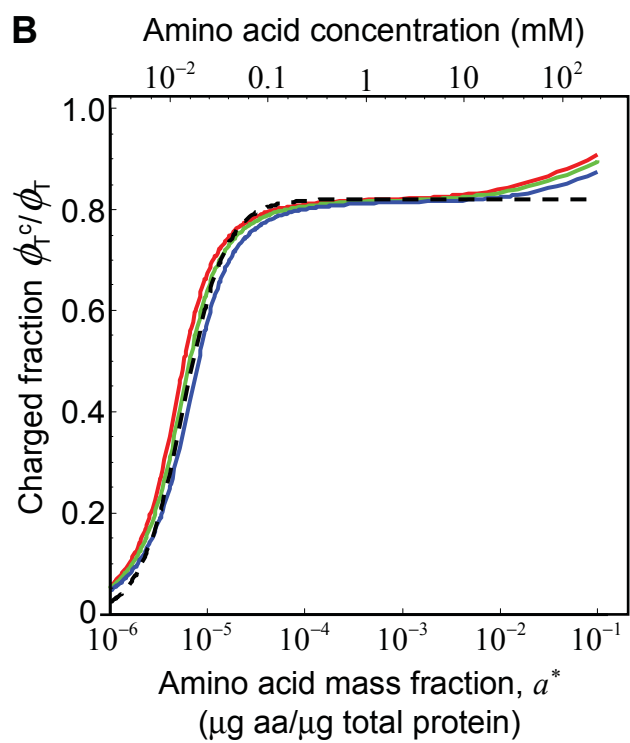

Supplement: Supplementary file 6 — Supplementary Figure S6 [file msb0010-0747-SD6.pdf]
