# Supplementary material for: Emergence of robust growth laws from optimal regulation of ribosome synthesis
Source: Mol Syst Biol. 2014 Aug 22;10(8):747. doi: 10.15252/msb.20145379 (PMC4299513; doi:10.15252/msb.20145379)
Supplement: Supplementary file 7 — Supplementary Information [file msb0010-0747-SD7.docx]

# Supplementary information

**Supplementary Figure S1 - Amino acid supply flux in media supporting slow growth.**

**A**. A recent study investigating the role of cyclic-AMP signaling in coordinating metabolism with the global gene expression and proteome partitioning lead to a refinement of the metabolic protein fraction into catabolic proteins with mass fraction $\varphi_{\text{C}}$ and anabolic proteins with mass fraction $\varphi_{\text{A}}$ (You et al, 2013). When amino acids are not supplied in the growth medium, amino acid supply is driven by anabolic proteins. For carbon-limited growth, the anabolic protein fraction shares the same growth dependence as the ribosomal proteins – collectively referred to as the RA-proteins with mass fraction $\varphi_{\text{RA}}=\varphi_{\text{R}}+\varphi_{\text{A}}$. The amino acid supply flux per mass $J_{a}^{in}/M$is proportional to the mass fraction of anabolic proteins $\varphi_{\text{A}}$,

$\frac{J_{a}^{in}}{M}=k_{A} \eta_{A} \varphi_{\text{A}}$.

As in the main text, $k_{A}$ characterizes the efficiency of the supply enzymes, and $\eta_{A}$ is the mass fraction of anabolic proteins devoted to amino acid biosynthesis.

**B**. Amino acids are synthesized from a pool of precursor metabolites (with mass fraction *K* ), with supply of precursors driven by catabolic proteins and proportional to $\varphi_{\text{C}}$,

$\frac{J_{K}^{in}}{M}=k_{C} \eta_{C} \varphi_{C}$.

The precursors $K$ are depleted via conversion into amino acids $a$ so that $J_{K}^{out}=J_{a}^{in}$, and the overall kinetics for precursor $K$ and amino acid $a$ dynamics are governed by,

$$\begin{matrix} \frac{da}{dt}=\eta_{A}k_{A}\varphi_{\text{A}}-\frac{J_{a}^{out}}{M}, \\ \frac{dK}{dt}=\eta_{C}k_{C}\varphi_{\text{C}}-\eta_{A}k_{A}\varphi_{\text{A}}. \end{matrix}$$

At steady-state, combining both equations to eliminate $\eta_{A} k_{A} \varphi_{\text{A}}$yields,

$\eta_{C} k_{C} \varphi_{\text{C}}=\frac{J_{a}^{out}}{M}$.

Protein synthesis consumes amino acids, and we identify the outflux $J_{a}^{out}/M$ with the rate of protein synthesis,

$\frac{J_{a}^{out}}{M}=\beta\times\gamma\left(\varphi_{\text{R}}-\varphi_{\text{R}}^{\min}\right)$,

where $\beta$ are the fraction of translation events that consume the growth-limiting amino acid. Combined with Eq. , this leads to a constraint linking the catabolic and ribosomal protein fractions,

$\frac{\eta_{C} k_{C}}{\beta} \varphi_{\text{C}}=\gamma\left(\varphi_{\text{R}}-\varphi_{\text{R}}^{\min}\right)$.

In carbon-poor medium, the catabolic protein fraction is synonymous with what is called the metabolic protein fraction ($\varphi_{\text{P}}$) in the main text, leading to a constraint identical to the steady-state of Eq. [10],

$\frac{\eta_{p} k_{p} }{\beta}\varphi_{\text{P}}=\gamma\left(\varphi_{\text{R}}-\varphi_{\text{R}}^{\min}\right)$.

The only difference between this case and the case derived for growth in amino acid-supplemented medium in the main text is that the proteome partitioning used to connect the amino acid flux constraint with the empirical growth relation (Eq. [2]) modifies the interpretation of the phenomenological parameter $\varphi_{\text{R}}^{\text{max}}$. The presence of the anabolic protein fraction, which follows the growth dependence of the ribosomal protein fraction, results in the partitioning,

$\varphi_{\text{P}}=\varphi_{\text{C}}=1-\varphi_{\text{Q}}-\varphi_{\text{RA}}=\varphi_{\text{R}}^{\text{max}}-\varphi_{\text{RA}}$,

where $\varphi_{\text{RA}}=\varphi_{\text{R}}+\varphi_{\text{A}}$. Writing the RA-protein fraction in terms of ribosomal protein fraction,

$\varphi_{\text{RA}}=\rho_{RA/R}\varphi_{\text{R}}$,

results in an equation similar to the empirical growth relation (Eq. [2]), but with a modified interpretation for the intercept at zero growth,

$\lambda=\nu\left(\frac{\varphi_{\text{R}}^{\text{max}}}{\rho_{RA/R}}-\varphi_{\text{R}}\right)$.

For growth limited by transport of a simple carbon source $c$, the efficacy of transport $k_{C}$can be written in a Michaelis-form,

$$k_{C}=k_{cat}\frac{c_{ex}}{c_{ex}+K_{M}}$$

where $c_{ex}$ is the external concentration, and $k_{cat}$ and $K_{M}$ characterize the speed and affinity of the transporter. With substitution into Eq. (with $\nu=\eta_{C}k_{cat}/\beta$), and using $\lambda=\gamma\left(\varphi_{\text{R}}-\varphi_{\text{R}}^{\text{min}}\right)$ to eliminate $\varphi_{\text{R}}$, yields a Monod relation for the growth rate $\lambda$(Monod, 1949),

$$\lambda=\left[\frac{\left(\varphi_{\text{R}}^{\text{max}}-\varphi_{\text{R}}^{\text{min}}\right)}{\frac{1}{\gamma}+\frac{1}{\nu}}\right]\frac{c_{ex}}{c_{ex}+\hat{K}_{M}}\equiv\lambda_{\infty}\frac{c_{ex}}{c_{ex}+\hat{K}_{M}},$$

where $\lambda_{\infty}$ is the growth rate in limit $c_{ex}\to\infty$. The *in vitro* Michaelis constant $K_{M}$ is apparently modified by the physiology of the organism to $\hat{K}_{M}=K_{M}/\left(1+\frac{\nu}{\gamma}\right)$ ; it now carries an explicit growth-medium dependence through the translation efficiency $\gamma$ and the nutrient efficiency $\nu$.

**Supplementary Figure S2 - The steady-state is a global attractor for the system.**

Combining the constraint on amino acid flux (Fig. 2A) with the supply-driven activation of ribosomal protein synthesis results in a dynamic equation for the internal amino acid level $a$,

$\frac{da}{dt}=\nu\left(a\right)\left[\varphi_{\text{R}}^{\text{max}}-\chi_{\text{R}}\left(a\right)\right]-\gamma\left(a\right)\left[\chi_{\text{R}}\left(a\right)-\varphi_{\text{R}}^{\text{min}}\right]$.

The amino acid supply rate $\nu\left(a\right)$ is a monotone decreasing function of the amino acid concentration $a$ mediated by negative feedback inhibition, whereas the elongation rate $\gamma\left(a\right)$ is a monotone increasing function of the amino acid concentration $a$, decreasing when amino acids become scarce due to insufficient tRNA charging. The ribosomal protein synthesis control function $\chi_{\text{R}}\left(a\right)$ is bound by the empirical constraint $\varphi_{\text{R}}^{\text{min}}\leq\chi_{\text{R}}\left(a\right)\leq\varphi_{\text{R}}^{\text{max}}$, and is assumed to be a monotone increasing function of the amino acid concentration $a$ mediated by ppGpp. Irrespective of the detailed form of these functions $\nu\left(a\right)$, $\gamma\left(a\right)$ and $\chi_{\text{R}}\left(a\right)$, monotonicity guarantees that the system has a single stable steady-state $a^{*}$, which is illustrated schematically in the figure. Plotting the individual flux contributions along the vertical axis as a function of the amino acid concentration $a$, the intersection of the supply flux (green line) with the consumption flux (red line) corresponds to the steady-state concentration $a^{*}$ at which $da/dt=0$ (dashed line). For amino acid concentrations below the steady-state, $a<a^{*}$, the supply flux exceeds the consumption flux and the amino acid level is increased to $a^{*}$ (region right of the dashed line). For amino acid concentrations above the steady-state, $a>a^{*}$, the opposite occurs; the consumption flux exceeds the supply flux and the amino acid level is decreased to $a^{*}$ (region left of the dashed line). In either case, $a^{*}$ is an attracting steady-state for the system.

**Supplementary Figure S3 – Estimates of amino acid pool size, $K_{\gamma}$ and $K_{\nu}$ in minimal media.**

A graphical representation of the data compiled in Table S1. Amino acid concentrations (blue bars) are compared with the tRNA-ligase affinity for their cognate amino acid (green bars), and the half-inhibition concentration for the major node of negative feedback inhibition of the biosynthetic operons (pink bars).

**Supplementary Figure S4 - Ternary complex-binding and translational elongation.**

**A.** To account for the role of ternary complex binding, we divide the ribosomal protein sector into two parts: a ribosome-affiliated protein mass fraction $\varphi{}_{\text{Rb}}$ and tRNA-affiliated protein fraction $\varphi_{\text{T}}$ (Klumpp et al, 2013),

$\varphi_{\text{R}}=\varphi_{\text{Rb}}+\varphi_{\text{T}}$.

Empirically, the ribosome-affiliated proteins $\varphi{}_{\text{Rb}}$ and the tRNA-affiliated proteins $\varphi_{\text{T}}$ are proportional to one-another in an approximately growth-independent manner (Howe & Hershey, 1983; Neidhardt et al, 1977; Pedersen et al, 1978),

$\varphi_{\text{T}}=\alpha\varphi_{\text{Rb}}$,

where $\alpha\approx0.6$ (Klumpp et al, 2013). As a consequence, $\varphi_{\text{T}}=\left(\alpha/1+\alpha\right)\varphi_{\text{R}}\approx0.38 \varphi_{\text{R}}$and $\varphi_{\text{Rb}}\approx0.62 \varphi_{\text{R}}$. This constant partitioning of the ribosomal protein fraction is achieved largely through co-expression from the same operons (Nomura et al, 1984), and appears to represent a near-optimal strategy to maintain rapid growth (Klumpp et al, 2013).

**B**. Peptide elongation is a multi-step process (see, for example, (Rodnina et al, 2005)). Here, we collect the binding of the ternary complex to the ribosome into a single step, and lump the remaining proofreading and peptidyl transfer together as a second step. Neglecting competition by non-cognate tRNA, binding of the ternary complex to the ribosome is well-approximated by a Michaelis-Menten expression, with ternary complex tRNA-affiliated protein mass fraction $\varphi_{\text{T}}^{c}$ playing the role of substrate and the active ribosome-affiliated protein mass fraction $\left(\varphi_{\text{Rb}}-\varphi_{\text{Rb}}^{\text{min}}\right)$ playing the role of enzyme (Ehrenberg & Kurland, 1984; Klumpp et al, 2013).

**Supplementary Figure S5 - Robust regulation of ribosome synthesis with explicit charged tRNA binding.**

**A**. The translation speed $\gamma\left(a^{*}\right)$ (Eq. ) normalized to its maximum $\gamma\left(a^{*}\to\infty\right)$ is shown as a function of the amino acid level for a range of nutrient quality: $\nu_{0}=2.5/h$ (red), $\nu_{0}=3.3/h$(blue), and $\nu_{0}=5.8/h$(green). High absolute abundance of tRNA-affiliated protein fraction $\varphi_{\text{T}}$ maintains rapid translation speed even when the charged fraction $\varepsilon\left(a^{*}\right)$ is not maximal (black dashed). For a charged fraction $\varepsilon\left(a^{*}\right)$ of 50% ($a^{*}=K_{\gamma}$), the translation speed $\gamma\left(a^{*}\right)$ is still about 80% of maximum, and does not drop to 50% until $a^{*}=K_{\gamma}/2$. For a Hill-type charging fraction $\varepsilon\left(a^{*}\right)$(Fig. S6; Eq. ), the effect of amino acid limitation on translation speed can be derived explicitly (Text S4).

**B.** When tRNA charging is included in the model, the optimality plateau in the ribosomal protein fraction $\varphi_{\text{R}}$ extends to the level of efficient elongation $K_{\gamma}$ (left dashed line). A large class of control functions $\chi_{\text{R}}\left(a\right)$ passes through the optimality plateau and provides robust autonomous optimal control over a range of nutrient environments. The light gray band corresponds to $\varphi_{\text{R}}^{\text{*}}$ within 10% of the optimum $\varphi_{\text{R}}^{\dagger}$. For comparison, the dark gray region corresponds to the same shown in Fig. 3. The colored lines and symbols correspond to those in Fig. 2. The proportionality between tRNA-affiliated protein and the ribosomal protein fraction, $\rho_{T/R}\approx0.38$**,** as well as the Michaelis constant for charged tRNA binding, $\phi_{\text{M}}/\varepsilon_{0}=0.029$, have been estimated previously (24). The remaining parameters are $\gamma_{0}=7.1/h$ and $\varphi_{\text{R}}^{\text{min}}=0.044$.

**Supplementary Figure S6 - Detailed model of tRNA charging.**

Elf and Ehrenberg have developed a mechanistic model for aminoacylation (Elf & Ehrenberg, 2005).

**A**. When co-regulation and constraints imposed through the coarse-grained proteome partition are included in the Elf and Ehrenberg model, the ‘optimality plateau’ in the steady-state ribosomal protein fraction $\varphi_{\text{R}}$ is similar to that observed in the simplified model presented in the Fig. S5.

**B**. The fraction of elongation factor proteins engaged in ternary complex, $\varphi_{\text{T}}^{\text{c}}/\varphi_{\text{T}}$ is approximately constant in the plateau region, and well-approximated by a growth-rate independent charged fraction $\varepsilon\left(a\right)$ as given in the Text S2, Eq. (black dashed line). Colored lines denote the nutrient quality of the medium: $\nu_{0}=2.5/h$ (red solid line), $\nu_{0}=3.3/h$ (blue solid line), and $\nu_{0}=5.8/h$ (green solid line). For illustration the black dashed line corresponds to Eq. with $\varepsilon_{0}=0.82$ and $K_{\gamma}=5.5\times10^{-6}$.

**Supplementary Table S1 - Amino acid pool-size, affinity for tRNA charging, affinity for feedback inhibition of biosynthesis (Figure S3)**

**Pools size and tRNA charging**: To facilitate comparison among different sources, the assay conditions were kept as similar as possible: 1) Pool size estimates come from exponentially growing cultures in glucose minimal media, with a doubling time of 60-75 minutes. 2) Amino acid binding affinity for the aminoacylation was determined for tRNA ligase isolated from *E. coli* using a diphosphate exchange assay done at 37°C, with pH=7.2-7.8. Table was compiled using the enzyme database BRENDA (Schomburg et al, 2002).

**Inhibition of biosynthesis**: Comparison among sources is difficult due to non-standardized assay conditions, and the combinatorial control of most synthesis pathways. Table was compiled with the aid of Ecocyc (Keseler et al, 2013).

| Amino acid | Pool size ($a^{*},$mM) | Ref | tRNA ligase  affinity ($K_{\gamma}$,mM) | Ref | Amino acid  affinity  ([gene]$K_{\nu}$,mM) | Ref | Fold Difference  ($K_{\nu}/K_{\gamma}$) |
| --- | --- | --- | --- | --- | --- | --- | --- |
| alanine | **2.60** | (Bennett et al, 2009) | **0.3** | (Filley & Hill, 1993) |  |  |  |
| valine | **4.00** | (Bennett et al, 2009) | **0.004** | (Tardif & Horowitz, 2004) |  |  |  |
| threonine | **0.18** | (Bennett et al, 2009) | **0.11** | (Sankaranarayanan et al, 2000) | [ThrA] **0.33** | (James & Viola, 2002) | 3 |
| leucine | **0.8** | (Maaløe, 1979) | **0.015** | (Lue & Kelley, 2005) |  |  |  |
| serine | **0.07** | (Bennett et al, 2009) | **0.064** | (Borel et al, 1994) | [SerA] **0.075** | (Sugimoto & Pizer, 1968) | 1.2 |
| proline | **0.36** | (Bennett et al, 2009) | **0.3** | (Stehlin et al, 1997) |  |  |  |
| aspartic acid | **4.20** | (Bennett et al, 2009) | **0.06** | (Martin et al, 1997) |  |  |  |
| methionine | **0.15 / 0.25** | (Bennett et al, 2009)/  (Maaløe, 1979) | **0.078** | (Armstrong & Fairfield, 1975) | [MetA] **0.1-4**  (synergistic with S-adenosylmethionine) | (Lee et al, 1966) | 1.3-51.3 |
| glutamic acid | **96.00** | (Bennett et al, 2009) | **0.056** | (Sekine et al, 1999) | [GltBD] **28** | (Miller & Stadtman, 1972) | 0.29 |
| glutamine | **3.80** | (Bennett et al, 2009) | **0.15** | (Kern et al, 1980) | [GlnA] **6**  (subject to cumulative feedback inhibition (Woolfolk & Stadtman, 1967)) | (van Heeswijk et al, 2013) | 40 |
| phenylalanine | **0.02** | (Bennett et al, 2009) | **0.01** | (Roy et al, 2004) | [PheA] **0.1** | (Dopheide et al, 1972) | 10 |
| tyrosine | **0.03** | (Bennett et al, 2009) | **0.005** | (Kiga et al, 2002) | [TyrA] **0.290** | (Turnbull et al, 1991) | 58 |
| lysine | **0.41 / 1.0** | (Bennett et al, 2009)/  (Maaløe, 1979) | **0.0026** | (Ataide & Ibba, 2004) | [LysC] **0.3**  [DapA] **0.25-1** | (Stadtman et al, 1961)  (Geng et al, 2013) | 115  96-385 |
| tryptophan | **0.01 / 0.07** | (Bennett et al, 2009)/(Maaløe, 1979) | **0.017** | (Zuniga et al, 2002) |  |  |  |
| asparagine | **0.51** | (Bennett et al, 2009) | **0.015** | (Madern et al, 1992) | [AsnA] **0.12** | (Cedar & Schwartz, 1969) | 8 |
| histidine | **0.07** | (Bennett et al, 2009) | **0.008** | (Ruhlmann et al, 1997) | [HisG] **0.38** | (Sterboul et al, 1977) | 47.5 |
| arginine | **0.57** | (Bennett et al, 2009) | **0.11** | (Eriani et al, 1990) | [ArgR] **0.1-0.3** | (Jin et al, 2005) | 0.9-2.7 |

**Supplementary Text S1** **- Additional optimization criteria on evolutionary timescales**

The analysis carried out in the main text treats the empirically-derived phenomenological parameters, e.g., the nutrient uptake rate for a given medium $\nu_{0}$ and the maximum elongation rate $\gamma_{0}$, as fixed parameters for a given strain. The optimization of growth-rate we discuss is on a time scale more rapid than the evolutionary time scales where these parameters can change as well, depending upon the ecological context. For example, adapting to growth in glycerol, Herring *et al.* (Herring et al, 2006) were able to corroborate growth rate increase with increased activity of glycerol processing enzymes (in the phenomenological model, this would correspond to an increase in $\nu_{0}$). The effective peptide elongation rate $\gamma_{0}$ is a coarse-grained representation of a multistep process that includes kinetic proofreading and peptidyl transfer (Rodnina et al, 2005). There are physical limitations on how rapidly amino acids can be polymerized into polypeptide chains, but there is also a biological question of translation fidelity (Ehrenberg & Kurland, 1984; Okamoto & Savageau, 1984). Balancing the competing demand for rapid protein synthesis and fidelity of translation results in selective pressure operating not only on $\gamma_{0}$, but on the Michaelis constant for ternary complex-ribosome binding $\phi_{\text{M}}$ and the partitioning of the ribosomal protein fraction (the parameter $\alpha$ in Fig. S4) (Klumpp et al, 2013).

**Supplementary Text S2 - Incorporating charged tRNA binding**

The simple expression for the amino acid-dependent elongation rate $\gamma\left(a^{*}\right)$, Eq. in the main text, tacitly assumes that a reduction in the free amino acid pool directly attenuates the maximum elongation rate. In reality, the interplay between amino acid abundance and peptide elongation is more complex. Amino acids are supplied to the elongating ribosome through the binding of ternary complexes (EF-Tu-GTP-aa-tRNA) that include elongation factor EF-Tu, GTP and charged tRNA (Nierhaus, 2004). To emphasize the role of ternary complex binding plays in protein mass accumulation and optimal growth-rate regulation, it is useful to further partition the ribosomal protein mass fraction $\varphi_{\text{R}}$ to distinguish proteins associated with peptide elongation ($\varphi_{\text{Rb}}$) from those associated with tRNA charging and transport to the ribosome ($\varphi_{\text{T}}$) (Fig. S4). We will first address how a linear relation between the ribosomal protein fraction and growth rate arises from the resulting more complicated model, and then examine how the optimality plateau is extended.

Peptide elongation is a multi-step process that includes ternary complex binding, tRNA accommodation, proofreading and peptidyl transfer (Rodnina et al, 2005; Wintermeyer et al, 2004). Here, we use a coarse-grained description based on Michaelis-Menten kinetics (Ehrenberg & Kurland, 1984; Klumpp et al, 2013), which consists of two steps, reversible ternary complex binding and an irreversible peptide elongation step, driven by GTP hydrolysis. The latter step includes proofreading and peptidyl transfer, which are summarized by an effective rate $\gamma_{0}$. The effective elongation rate $\gamma$ is then written,

$\gamma=\gamma_{0}\frac{\varphi_{\text{T}}}{\varphi_{\text{T}}+\phi_{\text{M}}}$.

The Michaelis constant $\phi_{\text{M}}=0.029$ is close to the minimum imposed by diffusion (Klumpp et al, 2013); to maximize the translation speed, the tRNA-affiliated protein fraction $\varphi_{\text{T}}$ must be kept larger than $\phi_{\text{M}}$. This is achieved by expressing the tRNA-affiliated proteins $\varphi_{\text{T}}$ to a molar ratio of about 6:1 with ribosome-affiliated proteins $\varphi_{\text{Rb}}$, maintained in an approximately growth rate-independent manner through co-expression from the same operons (Nomura et al, 1984). High expression of tRNA-affiliated proteins $\varphi_{\text{T}}$ and their growth-rate independent proportionality to the ribosomal proteins $\varphi_{\text{R}}$ together result in a linear relation between growth rate and ribosomal protein fraction in balanced growth, despite the apparent complexity of the expression for translation speed, Eq. . In the limit that $\varphi_{\text{T}}>>\phi_{\text{M}}$, the translation speed is $\gamma\approx\gamma_{0}\left[1-\phi_{\text{M}}/\varphi_{\text{T}}\right]$, and the rate of protein mass accumulation in exponential steady state, Eq. in the main text, becomes,

$$\lambda\approx\gamma_{0}\left[1-\frac{\phi_{\text{M}}}{\varphi_{\text{T}}}\right]\left(\varphi_{\text{R}}-\varphi_{\text{R}}^{\text{min}}\right),$$

or, upon re-arranging to obtain an explicit expression for the ribosomal protein fraction that is linear in growth rate $\lambda$ (Klumpp et al, 2013),

$\varphi_{\text{R}}\approx\left[\varphi_{\text{R}}^{\text{min}}+\frac{\phi_{\text{M}}}{\alpha/\left(1+\alpha\right)}\right]+\frac{\lambda}{\gamma_{0}}$,

where the term in square brackets is approximately constant due to the proportionality between the tRNA-affiliated proteins and ribosomal proteins, $\varphi_{\text{T}}=\alpha/\left(1+\alpha\right) \varphi_{\text{R}}=0.38 \varphi_{\text{R}}$ (Fig S4).

Implicit in the preceding analysis is the assumption that a constant fraction of the tRNA-affiliated proteins are in charged complex. The fraction of tRNA-affiliated proteins in charged complex, $\varepsilon\left(a^{*}\right)$, will carry amino acid dependence through aminoacylation of the tRNA, and the elongation speed is written,

$\gamma\left(a^{*}\right)=\gamma_{0}\frac{\varepsilon\left(a^{*}\right)\varphi_{\text{T}}}{\varepsilon\left(a^{*}\right)\varphi_{\text{T}}+\phi_{\text{M}}}$.

The linear relation between ribosomal protein fraction and the growth rate (Eq. ) is then obtained in the limit $\varepsilon\left(a^{*}\right)\varphi_{\text{T}}>>\phi_{\text{M}}$. By keeping the absolute abundance of tRNA-affiliated protein high $\varphi_{\text{T}}$, a reduced charged fraction $\varepsilon\left(a^{*}\right)$ still results in a linear relation between the ribosomal protein fraction and the growth rate. As we now show, high absolute abundance of tRNA-affiliated proteins leads to an enlarged optimality plateau, and consequently more robust growth-rate regulation.

A detailed model of aminoacylation ((Elf & Ehrenberg, 2005); Text S3 and Fig. S6) suggests that the charging fraction $\varepsilon\left(a^{*}\right)$ is well-characterized by a simple sigmoidal form,

$\varepsilon\left(a^{*}\right)=\varepsilon_{0}\frac{\left(a^{*}/K_{\gamma}\right)^{2}}{1+\left(a^{*}/K_{\gamma}\right)^{2}}$.

Here, $\varepsilon_{0}$ is the maximum charged fraction (for amino acids for which this has been measured, $\varepsilon_{0}\approx0.7-0.8$ (Sorensen, 2001)). The amino acid level for efficient charging $K_{\gamma}$ is as described in the main text, and is taken to be the affinity of tRNA ligase for the cognate amino acid (Table S1 and Fig. S3). Using Eqs. and for $\gamma\left(a^{*}\right)$ in the mass accumulation constraint, along with the nutrient flux constraint, yields the steady-state ribosomal protein fraction $\varphi_{\text{R}}^{\text{*}}$, internal amino acid pool $a^{*}$ and growth rate $\lambda$. Figure S5A illustrates the resilience of the translation speed to a drop in charged fraction $\varepsilon\left(a^{*}\right)$: The normalized translation speed $\gamma\left(a^{*}\right)/\gamma\left(a^{*}\to\infty\right)$ is shown as a function of the steady-state amino acid level for a variety of nutrient (colored lines, Fig. S5A). For comparison, the black dashed line is the charged fraction. By keeping the ternary complex proteins in high-abundance, the translation speed becomes more tolerant to substrate limitation: a drop in the charged fraction of 50% (vertical black line) corresponds to a 20% drop in the translation speed. Figure S5B shows how the optimality plateau in $a^{*}\left(\varphi_{\text{R}}\right)$ is widened by the inclusion of tRNA charging, particularly in favorable growth environments where both $\varphi_{\text{T}}$ and $\varphi_{\text{R}}$ are large (i.e., large $\nu_{0}$ - green line; Fig. S5B). With a widened plateau, the control function regulating ribosomal protein synthesis,$\chi_{\text{R}}\left(a\right)$, can be drawn from a large class of functions and still provides near-optimum partitioning of the proteome $\varphi_{\mathrm{R}}^{*}\approx\varphi_{\text{R}}^{\dagger}$. The light gray band in Fig. S5B correspond to the domain of the control function $\chi_{\text{R}}\left(a\right)$ that determines the ribosomal protein fraction $\varphi_{\text{R}}$ to within $\pm10\%$ of the optimum $\varphi_{\text{R}}^{\dagger}$; for comparison, the dark gray band from Fig. 3C is included as a hatched region to illustrate the contribution of a more detailed model of peptide elongation. In particular, steady-state amino acid levels close to that of efficient tRNA charging, $a*\approx K_{\gamma}$(left dashed line; Fig. S5B), now belong to the regime of optimal growth in contrast to the simplified model presented in the main text.

**Supplementary Text S3** **- Detailed model of tRNA charging by Elf & Ehrenberg**

Elf and Ehrenberg consider a more detailed scheme for amino acid supply, aminoacylation and protein synthesis (Elf & Ehrenberg, 2005),

$$\begin{matrix} & r_{1} & & r_{2} & & r_{3} & \\ \text{Nutrient} & \xrightarrow{} & a & \xrightarrow{} & \varphi_{T}^{c} & \xrightarrow{} & \text{Protein,} \end{matrix}$$

where $a$ is the amino acid level, and $\varphi_{T}^{c}$ the protein fraction engaged in ternary complex. By including constraints imposed by proteome partition and co-regulation of translation-affiliated proteins, we can recover the ‘optimality plateau’ described in the main text, and in so doing validate the charged fraction $\varepsilon\left(a\right)$ shown in Eq. by comparing with their expression for the protein fraction engaged in ternary complex $\varphi_{\text{T}}^{c}$, and setting $\varepsilon\left(a\right)=\varphi_{\text{T}}^{\text{c}}/\varphi_{\text{T}}$.

As in the main text, the Elf and Ehrenberg model assumes the supply of amino acids is proportional to the metabolic protein fraction $\varphi_{\text{P}}=\varphi_{\text{R}}^{\text{max}}-\varphi_{\text{R}}$, with feedback inhibition,

$r_{1}=\frac{\nu_{0}}{1+\left(a/K_{\nu}\right)}\left(\varphi_{\text{R}}^{\max}-\varphi_{\text{R}}\right)$.

After coarse-graining over different amino acid species, the rate of total protein synthesis, is similar in both models,

$r_{3}=\gamma_{0}\frac{\varphi_{\text{T}}^{c}}{\varphi_{\text{T}}^{c}+\phi_{\text{M}}}\left(\varphi_{\text{R}}-\varphi_{\text{R}}^{\text{min}}\right)$.

Under the assumption that ATP saturates the synthetase, tRNA rapidly equilibrates with the synthetase and that the aminoacyl-adenylate is in stable complex, the rate of consumption of amino acid via aminoacylation is,

$r_{2}=k_{2}\varphi_{\text{TS}}\frac{a}{\frac{k_{2}}{k_{0}}+a\left(1+\frac{K_{s}}{\varphi_{\text{T}}-\varphi_{\text{T}}^{c}}\right)}$,

where $\varphi_{\text{TS}}$ is the mass fraction of synthetase proteins, and $\varphi_{\text{T}}$ is the mass fraction of elongation factors. It is the effect of this auxiliary aminoacylation step that we represent by the charged fraction $\varepsilon\left(a\right)$in the main text.

In the background of Elf and Ehrenberg’s analysis is an implicit coupling between the abundances of metabolic proteins $\varphi_{\text{P}}$, the synthetase enzymes $\varphi_{\text{TS}}$ and elongation factors $\varphi_{\text{T}}$ via the overall proteome constraint $\varphi_{\text{R}}=\varphi_{\text{R}}^{\text{max}}-\varphi_{\text{P}}$ and co-expression of the protein synthesis machinery, $\varphi_{\text{R}}=\varphi_{\text{Rb}}+\varphi_{\text{T}}+\varphi_{\text{Ts}}$ (in Text S2, synthetase enzymes and elongation factors are lumped together in$\varphi_{\text{T}}$). The synthetase and elongation factors are co-expressed with ribosomal proteins, and thus proportional to the ribosomal protein fraction $\varphi_{\text{R}}$. It has been estimated previously that $\varphi_{\text{TS}}\approx0.14\varphi_{\text{R}}$ and $\varphi_{\text{T}}\approx0.24\varphi_{\text{R}}$, approximately independent of the growth rate (Klumpp et al, 2013), [with the remainder of the ribosomal protein fraction devoted to translation-associated factors, $\varphi_{\text{Rb}}=0.62\varphi_{\text{R}}$; see Fig. S4]. Converting concentration to mass fraction, the kinetic constants from Elf and Ehrenberg are approximately$k_{2}/k_{0}=3.8\times10^{-5}$ and $K_{s}\approx\phi_{\text{M}}$. The rate $k_{2}=120h^{-1}$ has been increased two-fold from the estimate by Elf and Ehrenberg so that the charged fraction in the plateau $\varphi_{\text{T}}^{\text{c}}/\varphi_{\text{T}}\approx0.8$ in accordance with measurements by Sorensen (Sorensen, 2001).

The steady-state conditions on the system are a flux balance,

$$\begin{matrix} r_{1}-r_{2}=0, \\ r_{2}-r_{3}=0, \end{matrix}$$

that are solved to obtain a parametric expression for $\varphi_{\text{R}}^{\text{*}}\left(a\right)$ and $\varphi_{\text{T}}^{\text{c,*}}\left(a\right)$ subject to the constraints that $0\leq\varphi_{\text{R}}\leq\varphi_{\text{R}}^{\text{max}}$ and $0\leq\varphi_{\text{T}}^{\text{c}}\leq\varphi_{\text{T}}$ (Fig. S6). The ‘optimality plateau’ in $\varphi_{\text{R}}^{\text{*}}\left(a\right)$ is evident in Fig. S6A, with colors corresponding to the solid curves in Fig. S5B. The expression for $\varphi_{\text{T}}^{\text{c,*}}\left(a\right)$ obtained from the model by Elf and Ehrenberg (Fig. S6B) is well-approximated by assuming a charged fraction $\varepsilon\left(a\right)=\varphi_{\text{T}}^{\text{c}}/\varphi_{\text{T}}$ as in Text S2, Eq. (Fig. S6B, black dashed line).

**Supplementary Text S4** **- Effective reduction in $K_{\gamma}$ due to charged tRNA binding**

The elongation rate depends upon the tRNA charging level through binding of the ternary complex to the elongating ribosome. The absolute concentration scale characterizing saturation of the elongating ribosome with ternary complex is given by the Michaelis constant $\phi_{M}$. Irrespective of relative charging levels $\varepsilon\left(a\right)$, if the absolute level of ternary complex exceeds the dissociation constant, $\varepsilon\left(a\right)\varphi_{\text{T}}>>\phi_{\text{M}}$, then the elongation rate will be close to maximal. In this way, by coordinating the expression of tRNA-affiliated and ribosome-affiliated proteins, the bacterium builds in a tolerance for perturbation in tRNA charging by maintaining a high absolute abundance of tRNA-affiliated proteins.

If the charged fraction $\varepsilon\left(a\right)$ is approximated by Hill-function form, Eq. , the effect on the elongation rate can be computed explicitly, and simply results in a re-scaling of the Michaelis constants $\phi_{M}$ and $K_{\gamma}$,

,

where $\hat{\phi}_{M}=\phi_{M}/\varepsilon_{0}$ is the Michaelis constant for ternary complex-ribosome binding scaled relative the maximum charged fraction $\varepsilon_{0}$, and the effective amino acid level for efficient peptide elongation, $\hat{K}_{\gamma}$, has been reduced from $K_{\gamma}$ to,

$\hat{K}_{\gamma}=K_{\gamma}\sqrt{\frac{1}{1+\varphi_{\text{T}}/\hat{\phi}_{M}}}$,

In the limit that $\varepsilon_{0}\varphi_{\text{T}}\gg\phi_{\text{M}}$, the translation rate, Eq. , coincides with the simple model presented in Eq. of the main text, but with considerably reduced effective amino acid level for efficient peptide elongation $\hat{K}_{\gamma}$.

NOTE: All references cited here can be found in the Reference list of the main article.
